# N6-Adenosine Methylation in MiRNAs

**DOI:** 10.1371/journal.pone.0118438

**Published:** 2015-02-27

**Authors:** Tea Berulava, Sven Rahmann, Katrin Rademacher, Ludgar Klein-Hitpass, Bernhard Horsthemke

**Affiliations:** 1 Institute of Human Genetics, University Hospital Essen, University of Duisburg-Essen, Essen, Germany; 2 Genome Informatics, Institute of Human Genetics, University Hospital Essen, University of Duisburg-Essen, Essen, Germany; 3 Institute of Cell Biology (Tumor Research), University Hospital Essen, University of Duisburg-Essen, Essen, Germany; CNRS UMR7622 & University Paris 6 Pierre-et-Marie-Curie, FRANCE

## Abstract

Methylation of N6-adenosine (m6A) has been observed in many different classes of RNA, but its prevalence in microRNAs (miRNAs) has not yet been studied. Here we show that a knockdown of the m6A demethylase FTO affects the steady-state levels of several miRNAs. Moreover, RNA immunoprecipitation with an anti-m6A-antibody followed by RNA-seq revealed that a significant fraction of miRNAs contains m6A. By motif searches we have discovered consensus sequences discriminating between methylated and unmethylated miRNAs. The epigenetic modification of an epigenetic modifier as described here adds a new layer to the complexity of the posttranscriptional regulation of gene expression.

## Introduction

Methylation of N6-adenosine (m6A) occurs in rRNA, tRNA, snoRNA, mRNA and lncRNA [1–4]. Transcriptome-wide mapping studies have shown that m6A in mRNA is enriched in the vicinity of stop codons, in the 5’ and 3’ untranslated regions (5’UTRs and 3’UTRs) and within internal long exons [3,4]. m6A appears to target mRNA for degradation [5]. The great majority of 3’UTRs that contain m6A sites also contain miRNA binding sites, but these rarely overlap [4].

miRNAs comprise a class of small non-coding RNAs that regulate expression of genes at the posttranscriptional level and thereby influence fundamental biological processes including cellular differentiation, proliferation, and apoptosis [6,7]. Their aberrant expression has been associated with numerous human diseases [8–11]. miRNAs are first transcribed as long primary transcripts (pri-miRNA), which are processed into precursor hairpin intermediates (pre-miRNA) [6,7]. The precursor hairpin is exported from the nucleus into the cytoplasm and further cleaved to 19–27-nt long mature miRNAs through a complex process. The functional strand of the mature miRNAs is loaded into the RNA induced silencing complex (RISC), which targets specific mRNA causing mRNA cleavage, translational repression or deadenylation. Each miRNA can target many different mRNAs, and each mRNA can be regulated by many different miRNAs [6,7].

The biogenesis of miRNAs is tightly regulated at multiple levels, including miRNA transcription, processing, loading into the RISC complex and finally its decay. All these steps may be affected not only by changes in the levels of executor molecules, but also by modification(s) of the sequence and/or structure of miRNA itself. The relevance of these alterations, in particular miRNA tailing [12,13], RNA editing [14,15] and RNA O-methylation [16] has intensively been studied apart of N6-adenosine methylation, although it has recently been claimed that the tRNA cytosine-methyltransferase NSun2 can methylate miR-125b at adenosine residues [17]. Here we report a comprehensive study of m6A in miRNAs.

## Material and Methods

### Antibodies

For RNA immunoprecipitation we used the anti-m6A antibody that has been used by Meyer *et al*.[4] (Synaptic System, Goettingen, Germany). Mouse mABb IgG1 isotope control antibody was used to control for non-specific binding (Cell Signaling Technology, Danvers, MA, USA). The primary antibodies used for western blotting were mouse and rabbit anti-FTO (Abcam, Cambridge, UK; Epitomics, Burlingame CA, USA) and rabbit anti-GAPDH (Cell Signaling Technology, Danvers, MA, USA). Secondary antibodies were goat anti-mouse and anti-rabbit HRP (horseradish peroxidase) conjugated antibodies (Thermo Fisher Scientific, Waltham, MA, USA)

### Knockdown of *FTO* by siRNA transfection

Uninduced Flp-In 293 T-Rex cell derivatives FTO1C1, FTO2D4 and FTO3C3 cell lines [18] were used for *FTO* knockdown experiments. Cells were maintained in DMEM medium supplemented with 10% FCS and 1% PenStrep in a humidified incubator at 37°C supplied with 5% CO2. Commercially available siRNA designed for *FTO* was purchased from Origene (Rockville, MD, USA. Cat. No SR312322). The kit provided three different siRNAs, two designed for the 3’UTR and one for exon 3. A universal scrambled siRNA absent in human, mouse and rat genomes was used as a negative control (included in the kit). As a transfection reagent, lipofectamine 2000 (Invitrogen, Carlsbad, CA, USA) was used.

Cells were transfected following the standard protocol at 10 nM concentration of siRNAs. Briefly, cells were plated in 6-well plate one day before transfection. On day of transfection, the medium was changed, and 2 h later the formed complexes of siRNAs and lipofectamine were added to cells in a drop-like manner. RNA and protein were extracted after 48 h of transfection from both, *FTO* siRNA and scrambled siRNA transfected cells. Knockdown efficiency was examined by qRT-PCR and western blotting as described [18].

### Total RNA Preparation and miRNAs enrichment

The large and small RNA fractions from *FTO* knockdown were prepared using RNeasyPlus Universal mini kit (Qiagen, Hilden, Germany) following the instructions of the manufacturer. The small RNA fraction was eluted with the RNeasyMinElute Cleanup Kit (Qiagen, Hilden, Germany). Using this approach, two fractions of RNA were prepared: large RNAs above 200 nt (mRNA with ribosomal RNAs) and small RNAs less than 200 nt in size (enriched with miRNAs, transferring RNAs, 5S and 5.8S rRNAs). Samples were analyzed on Agilent 2100 Bioanalyzer (Agilent Technologies, Santa Clara, CA, United States). The DNase treated large RNA was used for qRT-PCR to analyze the levels of mRNA from several genes and primary transcripts of miRNAs, the small RNA fraction was used for immunoprecipitation and high-throughput sequencing and for qRT-PCR to determine expression levels of mature miRNAs.

### RNA immunoprecipitaion

For immunoprecipitation of RNA, two rounds using 5 μg of anti-m6A antibody and 4 μg of small RNA were performed. The reaction was carried out using the Immunoprecipitation Kit—Dynabeads Protein G with some modifications (Life Technologies, Carlsbad, CA, USA)[4]. First, the antibody was coupled to Dynabeads Protein G in 500 μl of Binding and Washing Solution for 3 hours at 4°C followed by 10 minutes incubation at room temperature. Beads were then washed three times in Washing Buffer. Small RNA was added to the antibody-coupled beads in IP buffer (140 mM NaCl, 10 nM sodium phosphate, 0.05% Triton-X) supplemented with RNase inhibitor—Superase-In (Life Technologies, Carlsbad, CA, USA) and kept on the rotating platform at 4°C overnight. On the next morning beads were washed 5 times with IP buffer. Finally beads were treated with 250 μl of Elution Buffer (5 mM Tris pH 7.5, 1 mM EDTA, 0.05% SDS) supplemented with 2.1 μl Proteinase K (Invitrogen, 20 mg/ml) for 1.5 h on a heating block at 50°C at 1100 rpm. Immunoprecipitated RNA was recovered with Trizol LS reagent following the instructions from the manufacturer. As a control, immunoprecipitation was performed using IgG instead of anti-m6A antibody. The rest of experimental parameters were kept identical.

### Illumina miRNA sequencing

TruSeq Small RNA Sample Preparation Guide was used for cDNA synthesis and library preparation (Illumina, San Diego, CA, USA). The protocol was strictly followed. Briefly, 3’ and 5’ adapters were ligated to the samples, followed by reverse transcription and amplification with primers containing the unique indices for different samples. Amplified cDNA constructs were purified using Ampure XP beads. DNA was loaded into two lanes at 5 pM and 10 pM concentrations and subjected to Illumina sequencing. Demultiplexing was done using Illumina CASAVA 1.8.2.

### Sequence Analysis

#### Analysis Pipeline and software

The whole analysis pipeline was implemented as a Snakemake workflow to keep it reproducible [19]. Sequence matching and counting was done with the mamaslemonpy software that runs under Python 3 and is available from the Python Package Index (PyPI).

#### Adapter trimming and read classification

As mature miRNAs are shorter than the read length (16–28 bp vs. 50 bp), a typical miRNA read contains part of the adapter sequence (5′-TGGAATTCTCGGGTGCCAAGG-3′). Therefore adapter sequences are searched for and removed with cutadapt [20]. Cutadapt removes adapter sequences from high-throughput sequencing reads, requiring 4 bp overlap to identify the adapter and allowing a 20% error rate for the adapter sequence. Remaining reads shorter than 16 bp were discarded; reads between 16 bp and 28 bp in length were considered as candidate miRNA reads.

#### Identification and counting of mature miRNAs

The sequence reads were matched against the mature human miRNAs from miRBase release 20 of June 2013, which was the current release at the time of analysis [21]. For analyzing the *FTO* knockdown RNA-seq data, the *FTO* specific siRNA (SR312322A–gcagcugaaauauccuaaacuaatt, SR312322A_rc–uuaguuuaggauauuucagcugc, SR312322B–agacagaacuuagagacaucccagt, SR312322B_rc–acugggaugucucuaaguucugucu, SR312322C–agcgugugacaaagccuaaccuact and SR312322C _rc–aguagguuaggcuuugucacacgcu) and scrambled RNA (Scrambled–cguuaaucgcguauaauacgcguat and Scrambled_rc—auacgcguauuauacgcgauuaacg) sequences were added to the miRNA reference sequence. Entries with exactly the same sequence were collapsed into a single one. For filtering out the siRNAs, which are also represented in the library, their sequences were added to the set of reference sequences. Matching and identification of the appropriate reference for each read was done based on maximal exact matches (MEMs) [22]. In short, a MEM of length L with some reference sequence s starts at position p of a given read r if and only if the substring of length L at position p of r is equal to some substring of s, and this condition is violated if the match were extended in r to the left of position p or to the right of position p+L-1. For candidate miRNA and pre-miRNA reads, each MEM had to be of length at least 7, and all such MEMs had to cover at least 17 bp of the read. In the rare case where more than one reference satisfied these criteria, the reference with the most matching basepairs was considered the correct one. In the end, the number of reads that were successfully matched against each reference sequence were counted and tabulated. This resulted in a table of counts for each mature miRNA in each experiment.

The raw read numbers obtained from the matching step were normalized against the total counts of mapped miRNA reads per million as described [4,23–25]. miRNAs having at least 15 normalized reads in all three scrambled siRNA treated cell lines and fewer than 50% reads in the knockdown cells were defined as downregulated. miRNA having at least 15 normalized reads in all three knockdown cell lines and a fold-change >2 were defined as upregulated

For RNA samples immunoprecipitated with anti-m6A antibody (m6A samples) we applied stricter filtering options: (i) The cutoff was set to 100 normalized reads and (ii) at least 2 fold enrichment compared to samples immunoprecipitated with anti-IgG antibody (IgG samples) was required to consider a miRNA as methylated.

### Real-time PCR

RNA reverse transcribed into cDNA was used for quantitative Real-Time PCR (qRT-PCR) analyses. Dual color real-time PCR was performed on LightCycler480 System (Roche, Mannheim, Germany) using a Yellow-555-labeled universal reference *GAPDH* probe and the following FAM-labeled probes from the Universal Probe Library: #87 for *FTO* (forward primer 5’-gaaaatctggtggacaggtca-3’ and reverse primer 5’-cgagagagagtcatcctcactt-3’), #16 for *DICER1* (forward primer 5’-cagtcggcttcttcagtcg-3’ and reverse primer 5’-ggattccagtgatcctctgc-3’), #12 for *DROSHA* (forward primer 5’-tctctggaaaggtcctacaaaaa-3’ and reverse primer 5’-caggttcaggaacaaccgata-3’), #80 for *DGCR8* (forward primer 5’-tgcaaagatgaatccgttga-3’ and reverse primer 5’-agtaacttgctcaaagtcaaaacg-3’), #34 for *ADAR* (forward primer 5’-cggccaaggtttccagta-3’ and reverse primer 5’-cctgacttccgagatgcac-3’). LightCycler 480 probes master mix was used in reaction (Roche, Mannheim, Germany).

For mature miRNA the preformulated primer and probe sets in TaqMan Small RNA assay was used (Life Technologies, Carlsbad, CA, USA) following the cDNA generation with TaqMan MicroRNA Reverse Transcription kit (Life Technologies, Carlsbad, CA, USA). All reactions were performed according to the instructions from manufacturer. For primary transcripts predesigned primer and probe set was purchased from Life Technologies, and qRT-PCR was performed after reverse transcription of RNA into cDNA using GeneAmpl RNA PCR Kit and random hexamers (Applied Biosystems, Foster City, CA, USA).

### Unbiased search for discriminating motifs

To discover motifs that discriminate between the 239 immunoprecipitated miRNAs and 2555–239 = 2316 remaining miRNAs, we used two complementary methods.

Fist, we used the MoSDi software suite [26]. We generated all IUPAC consensus motifs of the forms 3–2–2–0, 4–2–2–1 and 5–3–2–1, where the form k-x-y-n means motifs of length k with at most x degenerate characters from the set {R, Y, K, M, W, S}, at most y degenerate characters from the set {B, D, H, V} and at most n Ns. The resulting motifs spaces contain 2466 (3–2–2–0), 37250 (4–2–2–1) and 571906 (5–3–2–1) motifs, respectively. We estimated a background text model from the k-mers of the 2316 remaining RNAs (k = 3, 4, 5 according to the motif space) and counted in how many of the 239 immunoprecipitated miRNAs each motif occurred. From the occurrence count and background model, MoSDi computes a p-value for each motif as described in detail previously [26].

Second, we used the counts of sequences with and without each motif in the immunoprecipitated and remaining sequences to form a 2x2 contingency table and obtained a p-value for each motif with Fisher’s exact test for independence between motif and the property of being pulled down. We used the Python 3.4 implementation of scipy.stats.fisher_exact, scipy version 0.13.3. Although both methods are based on complementary techniques (MoSDi is text-model based, whereas the other is test-based), we found the results to be highly similar, although the motifs slightly differ in rank when sorted by their p-value.

### Statistical analysis

The J.P. Royston version of the Shapiro-Wilk test was used to test the data for normality. The dependency of log2fc level after knockdown and log2f enrichment was analysed using the Pearson Product-Moment Correlation Coefficient for two sets of values.

## Results

**Fig 1:**
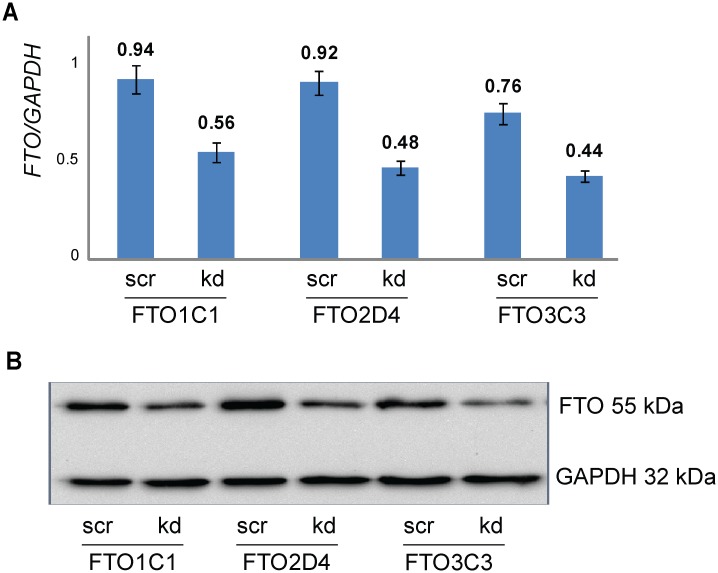
*FTO* knockdown in HEK293 cell clones FTO1C1, FTO2D4 and FTO3C3. A) The levels of *FTO* transcripts were investigated by qRT-PCR. *GAPDH* was used as a reference gene. Mean ± SD for three pairs of scrambled (scr) and *FTO* specific siRNAs treated cells are shown. B) Reduced protein levels were revealed by Western blot in all three cell lines transfected with siRNAs targeting FTO mRNA. Exemplary photo is depicted.

**Fig 2:**
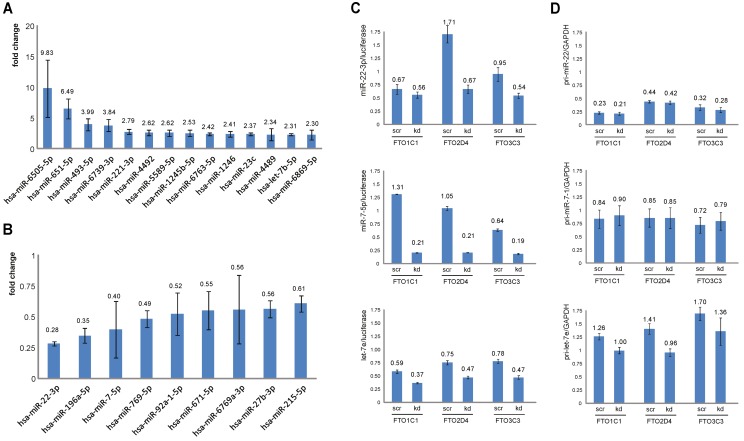
Deregulation of miRNAs in *FTO* knockdown cells. Mature miRNAs showing increased (A) and decreased (B) steady state levels in *FTO* knockdown cells. Normalized RNA-seq read numbers of individual miRNAs in *FTO* knockdown and scrambled siRNA treated cells were compared. Mean ± SD of three independent experiments are depicted. For verification and further studies, qRT-PCR analyses of selected mature miRNAs (C) and primary miRNA transcripts (D) were performed. We did not use other small RNAs as a reference gene for measuring mature miRNAs levels (as suggested by Life Technologies), since depletion of *FTO* might have an impact on their levels. Therefore, luciferase RNA was used to generate a standard curve and added to the qRT-PCR assays. *GAPDH* was used as a reference gene for measuring primary miRNAs transcript levels. Mean ± SD from quadruplicates per assay for three independent cell lines (FTO1C1, FTO2D4 and FTO3C3) are depicted. kd, *FTO* specific siRNA treated cells, scr, scrambled siRNA treated cells.

First, we investigated whether there is a link between adenosine methylation and miRNA levels. For this, we knocked down the ubiquitously expressed m6A demethylase FTO in HEK293 cells essentially as described before [18]. The knockdown efficiency in three independent experiments was 53–68% as determined by qRT-PCR and Western blot analysis (Fig. 1A,B). The levels of miRNAs were determined by RNA-seq. In total, 876 miRNAs were found to be expressed in these cells (S1 Table). After 48 hours of knockdown, we observed significantly increased levels of 42 miRNAs (the top 10 miRNAs are shown in Fig. 2A) and significantly decreased levels of nine miRNAs (Fig. 2B) with >2-fold change. Using a lower threshold (>1.5-fold change), we observed 249 upregulated and 35 downregulated miRNAs (S2 Table). The deregulation of miRNAs is intriguing, because mature miRNAs are assumed to be very stable. As shown by other studies, the levels of pre- and primary transcripts of miRNAs are well-regulated and most miRNAs persist long after RNAi-mediated removal of miRNA processing enzymes [6].

To verify the RNA-seq results and to determine whether the knockdown of *FTO* also affected the levels of primary miRNA transcripts (pri-miRNA), we performed qRT-PCR on let-7b, miR-7–5p and miR-22–3p miRNAs in quadruplicate experiments. In addition to let-7b, we also analyzed let-7e, although it was reduced by only 21% (S2 Table), because in a previous study [18] we had observed reduced transcript levels of *LIN28B*, which is a negative regulator of this particular family of miRNAs [27]. We confirmed reduced levels of let-7e, miR-7–5p and miR-22–3p miRNAs (Fig. 2C), but not increased levels of let-7b (not shown). Thus, in this experiment, the let-7 miRNA levels do not appear to be regulated by a simple feedback loop between LIN28B and let-7.

**Fig 3:**
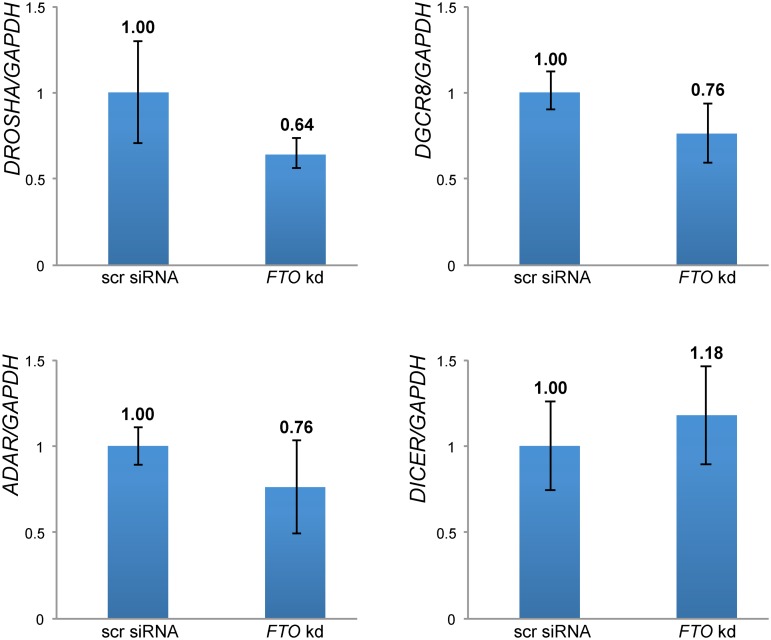
*K*nockdown of *FTO* does not significantly change mRNA levels of genes involved in miRNA biogenesis. The steady-state mRNA levels of *DICER*, *DROSHA*, *DGCR8* and *ADAR* were analyzed by qRT-PCR in cells treated with scrambled (scr) and *FTO-*specific siRNAs, respectively. *GAPDH* was used as a reference gene. The observed changes were not significant. Merged values of mean ± SD from triplicates per assay for the three independent cell lines FTO1C1, FTO2D4 and FTO3C3 are depicted. *FTO* kd, *FTO*-specific siRNA treated cells, scr siRNA, scrambled siRNA treated cells.

We did not observe any changes of the primary transcripts except of pri-let-7e, which showed a moderate downregulation (Fig. 2D). These results suggest that mainly the biogenesis or stability of the mature miRNAs was affected. One possibility is that the levels of factors involved in these processes are changed as a consequence of altered methylation levels of their transcripts. Indeed, the mRNAs of *DICER*, *DGCR8*, *ADAR*, *DROSHA*, and others carry m6A [3,4]. For testing this possibility, we measured the steady state levels of these mRNAs by qRT-PCR. As shown in Fig. 3, we observed small, but nonsignificant changes. These findings are in line with our previous microarray studies of HEK293 *FTO* knockdown cells, which had not revealed significant changes of these genes [18].

**Fig 4:**
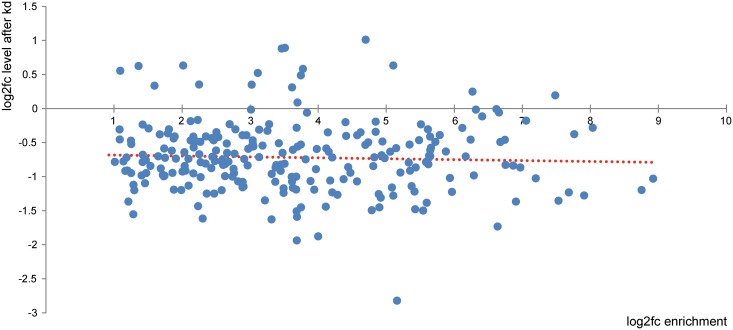
Effect of *FTO* knockdown on the steady state levels of methylated miRNAs. X-axis, log2 fold-changes (log2fc) of enrichment after imuunopreciptation with an anti-m6A antibody; y-axis, log2 fold-changes of steady state miRNA levels after *FTO* knockdown. The values of all 239 methylated miRNAs are shown. The red dotted line is the regression line.

A second possibility is that certain miRNAs are methylated at adenosine residues and that m6A affects miRNA biogenesis and/or stability. For a global view on m6A in miRNAs we grew large amounts of HEK293 cells and performed RNA immunoprecipitation followed by high-throughput sequencing (RIP-seq) in three independent experiments. For immunoprecipitation we used an anti-m6A antibody which has previously been used by Meyer et al. [4]. Since not all molecules of a specific miRNA may be methylated, we used a rather low fold-change threshold of 2, although we are aware that this may lead to the inclusion of false positive miRNAs. For reducing noise we only analyzed miRNAs with normalized read numbers of >100, although we probably miss miRNAs which are expressed and/or methylated at low levels in FTO containing cells. We found that 239 miRNAs were significantly enriched in the m6A samples compared to IgG samples. Many miRNAs (n = 124) were enriched >10-fold and 17 miRNAs were enriched >100-fold (Table 1 and S3 Table). The list of potentially methylated miRNAs includes miR-125b, which has recently been shown to be methylated [17]. Almost all these miRNAs (222/239 = 92%) were downregulated after *FTO* knockdown (Fig. 4), although in many cases the fold-changes of steady state levels were very small. The Shapiro-Wilk test shows that the distribution of the fold-changes significantly deviates from a normal distribution (p-value: <10^-8^). There was no significant correlation between fold change enrichment after immunoprecipitation and fold change of steady state levels after *FTO* knockdown (r = -0.047).

**Table 1:** miRNAs immunoprecipitated by the anti-m6A antibody.

Names	Fold enrichment	SD
hsa-miR-500a-3p	485.21	464.98
hsa-miR-103a-3p	431.65	260.54
hsa-miR-136–5p	262.50	202.93
hsa-miR-378a-3p	240.31	180.44
hsa-miR-1910–3p	217.40	77.61
hsa-miR-421	205.75	107.11
hsa-miR-28–5p	185.08	160.06
hsa-miR-3681–5p	179.17	54.76
hsa-miR-374b-5p	146.77	97.05
hsa-miR-6806–5p	133.11	32.84
hsa-miR-146b-5p	125.42	62.17
hsa-miR-320a	120.06	11.23
hsa-miR-548e-3p	116.69	68.97
hsa-miR-1255a	107.96	90.27
hsa-miR-4653–5p	107.21	9.94
hsa-miR-4298	102.29	33.42
hsa-miR-4791	100.95	47.98

Seventeen miRNAs with >100 fold enrichment after immunoprecipitation in m6A RNA samples compared to IgG RNA samples are listed. Means and standard deviations of fold enrichments in three independent experiments are given. The complete list of immunoprecipated miRNAs can be found in S3 Table.

Next we asked whether the immunoprecipitated miRNAs share a common sequence motif for the RNA adenosine methyltransferase in comparison to all 2555 miRNAs of miRBase version 20. Previous studies have shown that m6A occurs in mRNA and lncRNA within a highly conserved consensus sequence identified as RRACH (R = G or A; H = A, C or U) [3,4,28,29]. This sequence was also identified as a target site for methylation of the primary, precursor and mature miR-125b by the tRNA methyltransferase NSun2 [17]. Interestingly, in other classes of RNA, like rRNA, tRNA and snRNA, m6A is not necessarily found within the same consensus motif [30–32]. We found that 119/239 (49.7%) of the miRNAs contained the core RAC motif, whereas among all miRNAs, the numbers were 922/2555 (36.1%). Fisher’s exact test showed that the motif is very significantly enriched in the immunoprecipitated miRNAs (p-value: 5*10^-6^). For the more specific RRACH motif, the numbers were 78/239 (32.6%) vs. 535/2555 (20.9%), p-value 8*10^-6^, with the same conclusion.

For an unbiased search for the best discriminating IUPAC consensus motifs between immunoprecipitated miRNAs and the complementary set, we used two complementary methods: the MoSDi (Motif Search and Discovery) suite [26] and Fisher’s exact test on all IUPAC motifs of reasonable size with multiple testing correction (S4–S9 Tables). Both methods agreed on the results: For length 3, RAH (202/239 = 84.5% vs. 66.7%, Fisher p-value 10^-10^) and YGA (125/239 = 52.3% vs. 36.2%, p-value 10^-7^) together discriminated the immunoprecipitated miRNAs from the others (RAH contains RAC). For length 4, ADRA (133/239 = 55.6% vs. 32.1%, p-value 3*10^-15^) was the most discriminating motif (it overlaps with RAH and YGA). All Fisher p-values remain highly significant even after conservative Bonferroni multiple testing correction. The finding of the methyltransferase consensus sequence in our unbiased search supports the notion that certain miRNAs are methylated. It remains to be determined whether the other motifs are real or whether they reflect a bias of the antibody [33].

## Discussion

In a comprehensive analysis of human miRNAs by RNA immunopreciptation and high-throughput sequencing (RIP-seq) we have obtained evidence for the presence of m6A in miRNAs. The evidence is based (i) on the precipitation of a set of miRNAs with an anti-m6A-antibody and (ii) on the finding that these miRNAs are enriched for the methyltransferase consensus sequence RRACH. Unlike 5-methylcytosine (5mC), which can be identified at base-pair resolution by sodium bisulfite sequencing, a more direct detection of m6A is not possible at present and has to await the development of new sequencing techniques.

We have also found that the steady state level of certain miRNAs is affected by the level of the m6A demethylase FTO. The reason for this is not completely clear. Although our previous microarray studies [18] and the qRT-PCR studies performed here cannot completely exclude subtle changes in the concentration of miRNA processing factors, such changes are unlikely to lead to decreased levels of some miRNAs and increased levels of others.

Yuan et al. have shown that overexpression of the methyltransferase NSun2 inhibits the processing of miR-125b from pre-miR-125b2, but not from pre-miR-125b1. Silencing of NSun2 in cells with higher levels of pre-miR-125b2 than pre-miR-125b1 increased levels of mature miR-125b by ~2.2 fold [17]. In agreement with this finding we have found that the steady state levels of almost all potentially methylated miRNAs are reduced after knockdown of the m6A demethylase FTO. These findings support the notion that methylation affects the biogenesis and/or stability of miRNAs, but the situation is more complex: (i) we did not observe significant changes of the primary transcripts of several miRNAs tested, except for pri-let-7e, and (ii) many miRNAs are upregulated after *FTO* knockdown.

Increased miRNA levels after *FTO* knockdown are difficult to explain. They may result from increased stability of methylated miRNAs, although Yuan et al. have found no evidence for this in the case of miR-125b [17]. Another possibility is that certain miRNAs live longer as a consequence of attenuated gene silencing, caused by methylation of the miRNA [17] or by methylation of the target RNAs (as proposed by Meyer *et al*. [4]). Since many of the upregulated miRNAs are not methylated, the latter possibility may be true. In view of the fact that a miRNA typically targets a multitude of different mRNAs, this possibility is not easy to address experimentally. In any case, altered steady-state levels of miRNAs are likely to have functional consequences.

Single nucleotide polymorphisms in the *FTO* gene are associated with body weight regulation [34–37] and other, non-obese phenotypes [25,38–42]. Since these polymorphisms affect the steady levels of the *FTO* mRNA [43,44], it is likely that altered expression levels of *FTO* account for these associations. In view of the findings reported here it is tempting to speculate that part of the phenotypic effects of genetic variation at this locus is mediated through deregulated miRNAs. In fact, the deregulation of miRNAs has been implicated in obesity and other diseases (see for example [45]).

## Supporting Information

S1 Table List of miRNAs with at least 15 reads per million in all three input samples (FTO1C1, FTO2D4, FTOC3) (n = 876). scr, scrambled siRNA treated cells; kd, FTO knockdown cells.
(PDF)

S2 Table List of deregulated mature miRNAs in *FTO* knockdown cells. We detected 249 miRNAs with significantly increased levels (>1.5-fold change) (a) and 35 miRNAs with significantly decreased levels (b). Means and standard deviations of three compared pairs of scrambled and FTO specific siRNA treated cells are given.
(PDF)

S3 Table List of miRNAs significantly enriched (>2-fold change) in RNA samples immunoprecipitated with anti-me6A antibody compared to those captured by anti-IgG antibody (n = 239). Means of enrichment compared to IgG samples and their standard deviations are shown.
(PDF)

S4 Table List of the 46 motifs (3-mers) found to be significantly overrepresented in the immunoprecipitated miRNAs in comparison to the remaining miRNAs, as reported by MoSDi, sorted by score (decreasing). (PDF)

S5 Table List of the top 50 motifs (4-mers) found to be significantly overrepresented in the immunoprecipitated miRNAs in comparison to the remaining miRNAs, as reported by MoSDi, sorted by score (decreasing). (PDF)

S6 Table List of the top 50 motifs (5-mers) found to be significantly overrepresented in the immunoprecipitated miRNAs in comparison to the remaining miRNAs, as reported by MoSDi, sorted by score (decreasing). (PDF)

S7 Table List of the top 50 motifs (3-mers) found to be discriminating between immunoprecipitated miRNAs and remaining miRNAs by Fisher’s exact test on the number of sequences with and without the motif in immunoprecipitated and remaining miRNAs. (PDF)

S8 Table List of the top 50 motifs (4-mers) found to be discriminating between immunoprecipitated miRNAs and remaining miRNAs by Fisher’s exact test on the number of sequences with and without the motif in immunoprecipitated and remaining miRNAs. (PDF)

S9 Table List of the top 50 motifs found to be discriminating between immunoprecipitated miRNAs and remaining miRNAs by Fisher’s exact test on the number of sequences with and without the motif in immunoprecipitated and remaining miRNAs. (PDF)
