# Supplementary material for: N6-Adenosine Methylation in MiRNAs
Source: PLoS One. 2015 Feb 27;10(2):e0118438. doi: 10.1371/journal.pone.0118438 (PMC4344304; doi:10.1371/journal.pone.0118438)
Supplement: S1 Table — scr, scrambled siRNA treated cells; kd, FTO knockdown cells. (PDF) [file pone.0118438.s001.pdf]

Supplementary Table 1.

List of miRNAs with at least 15 reads per million in all three input samples (FTO1C1, FTO2D4, FTOC3) (n=876). scr, scrambled siRNA treated cells; kd, *FTO* knockdown cells.

	Name	**FTO1C1 scr**	**FTO2D4 scr**	**FTO3C3 scr**	**FTO1C1 kd**	**FTO2D4 kd**	**FTO3C3 kd**
1	hsa-let-7a-3p	70	90	65	58	56	34
2	hsa-let-7a-5p	5380	5426	8314	2854	3143	2650
3	hsa-let-7b-5p	38	47	38	38	52	52
4	hsa-let-7c-5p	178	113	213	118	95	153
5	hsa-let-7d-3p	35	32	92	18	22	33
6	hsa-let-7d-5p	20	18	16	6	7	9
7	hsa-let-7e-5p	233	175	192	90	95	102
8	hsa-let-7f-2-3p	18	19	16	21	14	15
9	hsa-let-7f-5p	11899	10729	9212	6801	5142	5993
10	hsa-let-7g-5p	347	369	351	239	212	236
11	hsa-let-7i-5p	290	351	200	242	221	217
12	hsa-miR-101-3p	2725	2738	2397	1635	1217	1013
13	hsa-miR-101-5p	234	188	154	154	124	142
14	hsa-miR-103a-3p	2231	1747	2171	950	740	992
15	hsa-miR-106b-3p	180	171	179	79	91	103
16	hsa-miR-106b-5p	693	734	683	393	317	343
17	hsa-miR-107	246	197	235	109	104	107
18	hsa-miR-10a-3p	161	139	140	74	74	67
19	hsa-miR-10a-5p	264567	227800	167204	101949	101929	114172
20	hsa-miR-10b-3p	77	77	67	46	48	45
21	hsa-miR-10b-5p	97412	75502	66125	35896	34826	42808
22	hsa-miR-1178-5p	20	19	21	13	15	13
23	hsa-miR-1180-3p	7590	7063	5843	2529	2019	1869
24	hsa-miR-1180-5p	23	29	27	18	15	15
25	hsa-miR-1181	25	38	39	27	30	25
26	hsa-miR-1184	383	464	449	390	260	347
27	hsa-miR-1185-1-3p	2158	2622	5764	1656	2167	1810
28	hsa-miR-1197	112	120	153	65	72	77
29	hsa-miR-1199-5p	24	18	34	15	12	17
30	hsa-miR-1200	49	36	34	24	21	21
31	hsa-miR-1203	19	15	25	17	18	17
32	hsa-miR-1204	260	225	773	105	127	169
33	hsa-miR-1205	15	16	17	12	11	10
34	hsa-miR-1207-3p	29	37	43	18	21	17
35	hsa-miR-122-3p	33	41	37	18	19	12

36	hsa-miR-1224-5p	23	28	25	19	17	17
37	hsa-miR-1231	23	24	16	14	18	14
38	hsa-miR-1234-3p	19	23	33	8	11	14
39	hsa-miR-1238-3p	22	20	34	10	14	12
40	hsa-miR-1238-5p	223	190	240	177	221	170
41	hsa-miR-124-5p	49	64	47	33	41	39
42	hsa-miR-1243	16	15	16	8	12	8
43	hsa-miR-1244	110	125	87	75	74	49
44	hsa-miR-1246	252	201	219	317	261	220
45	hsa-miR-1247-3p	24	24	22	11	10	14
46	hsa-miR-1247-5p	38	28	35	24	18	27
47	hsa-miR-1253	20	19	22	11	10	15
48	hsa-miR-1254	17	19	25	11	11	12
49	hsa-miR-1255a	26	24	20	12	13	14
50	hsa-miR-1256	234	330	267	154	265	238
51	hsa-miR-1258	1166	1003	764	996	914	1088
52	hsa-miR-125a-3p	21	19	19	12	13	11
53	hsa-miR-125a-5p	254	311	258	115	142	160
54	hsa-miR-125b-2-3p	344	263	354	149	110	164
55	hsa-miR-125b-5p	57	38	57	23	18	24
56	hsa-miR-126-3p	99	103	149	60	49	101
57	hsa-miR-126-5p	1341	1523	1954	950	684	1242
58	hsa-miR-1260b	109	220	159	116	138	95
59	hsa-miR-1261	3675	3309	2610	2811	2640	2576
60	hsa-miR-1262	31	22	28	15	23	19
61	hsa-miR-1266-5p	219	277	185	126	92	111
62	hsa-miR-127-3p	20	28	54	12	22	18
63	hsa-miR-1271-5p	98	65	40	31	30	41
64	hsa-miR-1272	69	78	62	56	62	55
65	hsa-miR-1273a	28	25	35	15	15	12
66	hsa-miR-1273c	71	45	81	36	51	42
67	hsa-miR-1273e	50	40	52	28	30	34
68	hsa-miR-1273g-3p	44	57	56	25	29	33
69	hsa-miR-1275	29	33	22	22	28	29
70	hsa-miR-1276	19	25	23	13	12	15
71	hsa-miR-1277-3p	482	478	405	331	222	201
72	hsa-miR-1277-5p	39	51	41	24	21	22
73	hsa-miR-128-1-5p	557	497	634	388	387	438
74	hsa-miR-128-3p	1050	846	1029	473	381	492
75	hsa-miR-1285-3p	202	182	159	88	88	100
76	hsa-miR-1286	89	83	66	29	24	26

77	hsa-miR-1287-5p	33	40	71	20	24	24
78	hsa-miR-1288-3p	17	24	24	14	15	18
79	hsa-miR-1288-5p	16	19	23	9	14	9
80	hsa-miR-1290	20	28	19	17	16	14
81	hsa-miR-1291	24	18	21	10	11	10
82	hsa-miR-1292-5p	16	17	15	9	8	10
83	hsa-miR-1296-5p	47	52	49	21	26	29
84	hsa-miR-1298-5p	25	21	26	16	14	12
85	hsa-miR-1299	31	28	26	17	17	23
86	hsa-miR-1301-3p	58	37	39	18	17	24
87	hsa-miR-1303	65	66	61	25	28	34
88	hsa-miR-1304-3p	115	82	107	50	44	46
89	hsa-miR-1305	51	49	33	24	18	15
90	hsa-miR-1306-3p	47	40	47	27	30	27
91	hsa-miR-1307-3p	194	181	244	91	87	109
92	hsa-miR-1307-5p	1294	1334	945	729	429	294
93	hsa-miR-130a-3p	283	321	315	126	134	177
94	hsa-miR-130b-3p	1641	1221	978	675	562	460
95	hsa-miR-130b-5p	358	253	291	141	114	134
96	hsa-miR-132-3p	52	38	56	23	25	37
97	hsa-miR-132-5p	23	23	27	13	15	16
98	hsa-miR-1322	39	35	35	23	24	23
99	hsa-miR-133a-5p	27	16	37	8	13	10
100	hsa-miR-1343-5p	28	24	30	21	21	19
101	hsa-miR-135a-5p	24	21	25	17	14	17
102	hsa-miR-136-5p	82	59	58	54	57	49
103	hsa-miR-137	69	82	83	61	62	61
104	hsa-miR-138-5p	42	37	57	18	17	25
105	hsa-miR-140-3p	294	357	306	106	135	123
106	hsa-miR-140-5p	46	51	41	36	25	32
107	hsa-miR-141-3p	45	42	35	24	26	17
108	hsa-miR-143-3p	42	46	59	22	27	29
109	hsa-miR-144-5p	21	32	20	16	19	9
110	hsa-miR-145-3p	42	37	30	34	27	31
111	hsa-miR-1468-3p	3887	2823	3231	1649	2035	2775
112	hsa-miR-146b-3p	25	21	22	12	11	10
113	hsa-miR-146b-5p	619	615	542	247	256	450
114	hsa-miR-148a-3p	34598	36667	30715	16107	12690	12542
115	hsa-miR-148a-5p	328	280	309	116	105	100
116	hsa-miR-148b-3p	3408	2766	3023	1551	1133	1343
117	hsa-miR-148b-5p	236	220	214	93	75	76

118	hsa-miR-149-3p	20	19	22	14	14	15
119	hsa-miR-149-5p	17	18	23	8	10	10
120	hsa-miR-151a-3p	5094	3475	3989	1743	1490	1850
121	hsa-miR-151a-5p	3106	2322	2394	1183	980	1224
122	hsa-miR-152-3p	53	58	43	53	50	44
123	hsa-miR-152-5p	66	45	63	35	28	55
124	hsa-miR-153-3p	32	33	24	19	20	17
125	hsa-miR-153-5p	641	461	581	336	350	537
126	hsa-miR-1537-5p	16	23	28	10	15	10
127	hsa-miR-1538	32	34	35	20	16	18
128	hsa-miR-155-3p	53	82	101	29	58	38
129	hsa-miR-15a-5p	571	412	553	317	198	257
130	hsa-miR-15b-3p	22	23	31	13	11	12
131	hsa-miR-15b-5p	200	170	218	64	66	97
132	hsa-miR-16-2-3p	85	87	91	60	53	53
133	hsa-miR-16-5p	14409	9703	14098	4976	3117	5616
134	hsa-miR-17-3p	255	172	237	155	77	125
135	hsa-miR-17-5p	1223	660	1427	572	311	700
136	hsa-miR-181a-2-3p	54	63	68	25	27	30
137	hsa-miR-181a-3p	138	174	129	53	74	54
138	hsa-miR-181a-5p	2067	2779	2120	952	1584	1125
139	hsa-miR-181b-3p	36	52	34	23	24	16
140	hsa-miR-181b-5p	953	923	996	310	387	358
141	hsa-miR-181c-3p	279	315	295	169	143	130
142	hsa-miR-181c-5p	694	755	733	468	514	469
143	hsa-miR-181d-3p	19	16	36	13	15	14
144	hsa-miR-181d-5p	313	347	328	181	166	168
145	hsa-miR-182-5p	30381	23326	25161	11652	11007	12131
146	hsa-miR-183-5p	7033	6248	5603	3920	3474	4101
147	hsa-miR-184	29	30	29	19	22	20
148	hsa-miR-185-3p	37	28	46	11	14	13
149	hsa-miR-186-5p	10964	8712	9262	4575	3547	4479
150	hsa-miR-187-3p	22	24	28	11	9	15
151	hsa-miR-187-5p	24	27	25	17	13	17
152	hsa-miR-18a-5p	408	273	461	223	103	219
153	hsa-miR-1908-3p	64	77	77	60	57	53
154	hsa-miR-1909-3p	1358	1143	2388	475	1012	938
155	hsa-miR-191-3p	22	17	24	14	12	15
156	hsa-miR-191-5p	6330	5313	5551	1946	2029	3295
157	hsa-miR-1910-3p	632	615	443	489	476	337
158	hsa-miR-1910-5p	19	19	27	8	6	9

159	hsa-miR-1912	29	21	35	16	17	15
160	hsa-miR-1913	76	86	66	58	59	45
161	hsa-miR-1914-5p	6522	7858	5251	4883	5092	4070
162	hsa-miR-192-5p	6659	6034	5366	2903	2199	2517
163	hsa-miR-193b-3p	34	48	43	17	23	16
164	hsa-miR-194-5p	111	101	111	56	43	53
165	hsa-miR-195-5p	37	31	35	10	12	13
166	hsa-miR-196a-3p	117	191	107	63	83	54
167	hsa-miR-196a-5p	2236	3284	1429	323	503	322
168	hsa-miR-196b-5p	3488	3214	2376	1810	1379	1302
169	hsa-miR-197-5p	31	50	38	28	33	30
170	hsa-miR-1972	18	21	21	8	11	11
171	hsa-miR-199a-3p___hsa-miR-199b-3p	41	25	39	13	15	17
172	hsa-miR-199b-5p	185	132	449	54	105	91
173	hsa-miR-19a-3p	1299	837	1167	864	375	623
174	hsa-miR-19b-3p	1472	952	1411	881	461	778
175	hsa-miR-200b-3p	377	218	312	157	177	159
176	hsa-miR-200b-5p	21	23	39	17	26	37
177	hsa-miR-200c-3p	118	81	121	87	67	85
178	hsa-miR-203a	52	37	58	33	33	35
179	hsa-miR-203b-3p	42	37	31	25	29	28
180	hsa-miR-2053	156	159	228	106	102	104
181	hsa-miR-2054	33	31	35	21	20	19
182	hsa-miR-20a-5p	891	495	1230	485	254	506
183	hsa-miR-21-3p	380	874	337	209	319	123
184	hsa-miR-21-5p	3593	7717	3331	1626	2444	1170
185	hsa-miR-210-3p	93	107	63	35	34	28
186	hsa-miR-2113	30	26	54	22	28	28
187	hsa-miR-2115-3p	96	106	83	64	80	75
188	hsa-miR-2116-3p	27	24	29	17	16	16
189	hsa-miR-2117	46	51	69	37	38	39
190	hsa-miR-212-5p	33	26	37	15	15	18
191	hsa-miR-215-5p	483	483	476	143	153	138
192	hsa-miR-218-1-3p	28	35	26	14	18	22
193	hsa-miR-218-5p	904	1004	1046	350	285	373
194	hsa-miR-219a-1-3p	20	17	16	11	11	8
195	hsa-miR-22-3p	5605	6804	5512	695	933	897
196	hsa-miR-221-3p	974	237	482	1476	284	688
197	hsa-miR-221-5p	637	119	245	220	45	151
198	hsa-miR-222-3p	4025	1270	3014	1709	501	1179

199	hsa-miR-222-5p	285	67	182	127	25	78
200	hsa-miR-223-3p	38	42	52	19	29	23
201	hsa-miR-224-5p	31	28	32	22	21	23
202	hsa-miR-2277-5p	67	77	70	32	27	28
203	hsa-miR-2355-3p	25	22	22	15	12	14
204	hsa-miR-2392	68	64	86	47	45	41
205	hsa-miR-23a-3p	31	31	27	11	16	19
206	hsa-miR-23b-3p	83	60	67	28	32	50
207	hsa-miR-23c	46	35	47	49	45	55
208	hsa-miR-24-2-5p	17	21	22	10	12	11
209	hsa-miR-24-3p	79	98	75	45	52	71
210	hsa-miR-2467-5p	65	59	44	30	18	19
211	hsa-miR-25-3p	17258	14189	15949	5015	4777	5610
212	hsa-miR-25-5p	117	101	110	46	44	57
213	hsa-miR-2681-3p	37	44	34	25	20	26
214	hsa-miR-26a-2-3p	27	22	27	19	17	14
215	hsa-miR-26a-5p	11583	11443	10906	5206	4599	5025
216	hsa-miR-26b-3p	47	34	51	28	18	32
217	hsa-miR-26b-5p	1622	1452	2133	1449	894	1951
218	hsa-miR-27a-3p	195	224	153	98	81	77
219	hsa-miR-27a-5p	25	34	30	18	25	17
220	hsa-miR-27b-3p	9697	9436	9203	2923	2441	2556
221	hsa-miR-27b-5p	121	102	88	53	52	48
222	hsa-miR-28-3p	1637	1547	1856	511	499	623
223	hsa-miR-28-5p	196	228	212	79	78	91
224	hsa-miR-2861	28	24	30	19	14	21
225	hsa-miR-296-3p	88	84	72	41	35	55
226	hsa-miR-298	114	137	181	100	99	157
227	hsa-miR-29a-3p	142	171	118	83	74	51
228	hsa-miR-29b-2-5p	24	17	23	14	15	23
229	hsa-miR-29b-3p	18	25	20	10	9	7
230	hsa-miR-29c-3p	133	172	143	68	69	60
231	hsa-miR-301a-3p	1073	1173	1068	756	699	597
232	hsa-miR-301a-5p	18	23	17	9	9	10
233	hsa-miR-301b	841	1033	541	606	634	314
234	hsa-miR-3065-5p	21	18	22	12	14	12
235	hsa-miR-3074-3p	24	22	15	12	11	11
236	hsa-miR-3074-5p	20	18	17	10	9	12
237	hsa-miR-30a-3p	236	260	251	258	261	283
238	hsa-miR-30a-5p	8667	11179	9448	3980	3875	3496
239	hsa-miR-30b-5p	232	236	254	110	102	100

240	hsa-miR-30c-1-3p	173	146	158	86	70	87
241	hsa-miR-30c-2-3p	50	37	42	19	21	23
242	hsa-miR-30c-5p	1047	1061	1463	436	401	506
243	hsa-miR-30d-3p	202	235	174	148	142	119
244	hsa-miR-30d-5p	2996	3451	3581	1375	1466	1684
245	hsa-miR-30e-3p	750	605	647	652	551	634
246	hsa-miR-30e-5p	13653	15078	13925	6206	4595	4892
247	hsa-miR-31-5p	455	504	514	171	164	186
248	hsa-miR-3121-3p	17	17	187	9	11	14
249	hsa-miR-3122	93	67	66	37	35	39
250	hsa-miR-3125	17	18	15	9	11	9
251	hsa-miR-3126-5p	21	18	21	11	16	14
252	hsa-miR-3128	24	17	27	9	14	9
253	hsa-miR-3129-5p	46	31	39	21	14	12
254	hsa-miR-3130-3p	16	17	20	10	10	10
255	hsa-miR-3135a	29	23	24	14	13	21
256	hsa-miR-3135b	28	16	39	8	9	11
257	hsa-miR-3137	38	34	33	27	27	26
258	hsa-miR-3138	19	21	17	11	12	15
259	hsa-miR-3142	23	21	50	7	12	15
260	hsa-miR-3143	27	27	30	20	14	13
261	hsa-miR-3144-3p	238	223	181	176	164	132
262	hsa-miR-3144-5p	46	37	50	23	22	21
263	hsa-miR-3147	59	49	61	26	28	30
264	hsa-miR-3150a-5p	28	29	38	18	17	17
265	hsa-miR-3150b-3p	21	24	19	16	17	19
266	hsa-miR-3158-3p	47	38	40	23	24	20
267	hsa-miR-3160-3p	27	20	22	13	13	17
268	hsa-miR-3161	28	27	28	19	22	19
269	hsa-miR-3164	25	24	28	17	16	21
270	hsa-miR-3168	168	191	510	158	116	211
271	hsa-miR-3170	17	16	27	9	11	14
272	hsa-miR-3171	20	18	25	11	10	10
273	hsa-miR-3173-5p	52	43	49	28	23	28
274	hsa-miR-3174	28	26	24	13	14	15
275	hsa-miR-3175	117	85	112	61	60	79
276	hsa-miR-3176	206	280	161	83	85	60
277	hsa-miR-3178	19	20	20	12	15	14
278	hsa-miR-3180-5p	25	31	44	17	18	21
279	hsa-miR-3181	572	425	774	360	301	579
280	hsa-miR-3182	1519	1503	1353	1209	1158	1105

281	hsa-miR-3183	37	33	38	28	22	27
282	hsa-miR-3184-5p	20	24	28	13	15	15
283	hsa-miR-3186-3p	17	17	32	10	13	14
284	hsa-miR-3189-3p	19	29	21	11	13	19
285	hsa-miR-3195	203	211	200	190	199	144
286	hsa-miR-3197	21	15	25	12	7	14
287	hsa-miR-32-3p	36	30	32	28	16	25
288	hsa-miR-32-5p	75	95	73	62	61	42
289	hsa-miR-3200-3p	15	16	17	9	11	9
290	hsa-miR-3200-5p	23	19	19	16	15	14
291	hsa-miR-3201	19	18	21	14	13	14
292	hsa-miR-3202	16	18	25	10	11	12
293	hsa-miR-320a	132	155	181	56	58	65
294	hsa-miR-324-3p	37	39	35	18	17	19
295	hsa-miR-324-5p	18	20	19	9	8	9
296	hsa-miR-329-5p	25	23	27	15	11	11
297	hsa-miR-330-3p	25	23	28	7	7	11
298	hsa-miR-330-5p	194	203	151	98	67	53
299	hsa-miR-331-3p	38	38	45	19	15	19
300	hsa-miR-331-5p	39	43	42	24	17	20
301	hsa-miR-335-3p	56	33	203	11	18	14
302	hsa-miR-337-3p	91	70	45	60	52	52
303	hsa-miR-338-3p	29	37	33	20	30	19
304	hsa-miR-338-5p	19	16	17	11	14	13
305	hsa-miR-339-3p	201	176	179	113	102	100
306	hsa-miR-339-5p	49	47	43	27	24	18
307	hsa-miR-33a-5p	63	56	75	30	19	18
308	hsa-miR-33b-3p	23	16	21	14	15	16
309	hsa-miR-33b-5p	19	27	17	13	9	7
310	hsa-miR-340-3p	111	84	104	51	38	48
311	hsa-miR-340-5p	1577	1494	1341	784	592	512
312	hsa-miR-342-3p	85	68	71	34	31	46
313	hsa-miR-345-3p	202	258	444	187	246	171
314	hsa-miR-345-5p	378	435	443	176	169	222
315	hsa-miR-34c-5p	37	43	44	21	19	18
316	hsa-miR-3529-3p	24	26	25	15	16	17
317	hsa-miR-3591-3p	16	35	20	11	13	10
318	hsa-miR-3605-5p	79	63	85	44	53	54
319	hsa-miR-3606-3p	719	845	617	408	582	414
320	hsa-miR-3607-5p	80	62	59	55	70	56
321	hsa-miR-3609	59	53	49	57	55	45

322	hsa-miR-361-5p	2611	1707	2309	1328	862	1100
323	hsa-miR-3611	96	76	110	59	69	70
324	hsa-miR-3613-3p	497	294	547	264	188	367
325	hsa-miR-3613-5p	83	57	61	57	37	57
326	hsa-miR-3614-3p	26	27	40	24	26	19
327	hsa-miR-3614-5p	1520	1453	894	1254	1016	774
328	hsa-miR-3615	95	69	108	49	45	57
329	hsa-miR-3617-3p	19	22	17	21	15	16
330	hsa-miR-3621	73	54	168	31	32	32
331	hsa-miR-3622b-3p	86	85	120	69	75	101
332	hsa-miR-363-3p	126	224	104	68	105	67
333	hsa-miR-363-5p	26	23	26	23	20	20
334	hsa-miR-3646	30	19	25	15	23	21
335	hsa-miR-3649	34	26	27	18	16	23
336	hsa-miR-3650	24	22	31	15	18	15
337	hsa-miR-3651	68	69	58	59	46	41
338	hsa-miR-3652	68	59	69	38	55	67
339	hsa-miR-3653	20	18	19	11	9	11
340	hsa-miR-365a-3p___hsa-miR-365b-3p	26	30	100	11	15	17
341	hsa-miR-3660	141	145	117	91	87	52
342	hsa-miR-3661	26	16	27	10	10	15
343	hsa-miR-3662	2618	2748	1650	1928	2167	1491
344	hsa-miR-3671	53	57	72	38	28	48
345	hsa-miR-3672	556	592	463	458	416	304
346	hsa-miR-3673	20	22	18	15	9	10
347	hsa-miR-3675-3p	25	23	30	15	15	18
348	hsa-miR-3677-3p	52	62	36	24	20	22
349	hsa-miR-3680-3p	19	18	27	16	19	20
350	hsa-miR-3680-5p	16	16	17	11	10	11
351	hsa-miR-3681-5p	26	31	19	23	35	26
352	hsa-miR-3682-3p	312	393	265	236	349	208
353	hsa-miR-3687	39	34	49	25	26	28
354	hsa-miR-3688-3p	16	23	21	8	10	10
355	hsa-miR-3688-5p	930	811	834	616	572	535
356	hsa-miR-3690	55	53	62	29	35	38
357	hsa-miR-3692-5p	16	21	17	7	11	13
358	hsa-miR-370-3p	1364	1376	1205	650	690	688
359	hsa-miR-370-5p	46	58	61	24	28	18
360	hsa-miR-371b-5p	40	34	46	33	23	31
361	hsa-miR-372-3p	49	43	46	31	32	42

362	hsa-miR-373-5p	39	58	68	32	41	36
363	hsa-miR-374a-3p	519	608	451	291	225	175
364	hsa-miR-374a-5p	301	344	259	157	135	139
365	hsa-miR-374b-3p	64	66	52	34	24	28
366	hsa-miR-374b-5p	129	126	120	58	61	65
367	hsa-miR-375	219	273	348	79	102	146
368	hsa-miR-376a-2-5p	66	79	66	40	35	37
369	hsa-miR-378a-3p	6756	4965	6945	2493	2197	2958
370	hsa-miR-378a-5p	21	19	28	11	13	11
371	hsa-miR-380-3p	71	111	71	37	41	31
372	hsa-miR-381-5p	29	18	24	14	17	14
373	hsa-miR-383-3p	17	20	15	10	12	9
374	hsa-miR-383-5p	25	19	15	11	11	9
375	hsa-miR-3908	68	58	197	32	40	43
376	hsa-miR-3913-5p	21	20	33	15	15	13
377	hsa-miR-3914	24	28	28	15	21	15
378	hsa-miR-3915	24	18	27	15	17	14
379	hsa-miR-3916	24	22	21	12	16	13
380	hsa-miR-3919	17	17	47	11	10	13
381	hsa-miR-3920	78	58	46	59	56	45
382	hsa-miR-3922-5p	310	307	252	168	185	137
383	hsa-miR-3925-5p	22	19	22	11	14	15
384	hsa-miR-3928-3p	35	29	28	16	17	20
385	hsa-miR-3928-5p	27	29	33	21	23	30
386	hsa-miR-3929	24	25	27	15	16	14
387	hsa-miR-3934-5p	120	100	115	97	95	76
388	hsa-miR-3937	463	603	364	427	323	198
389	hsa-miR-3938	28	28	24	16	15	12
390	hsa-miR-3939	25	16	22	9	9	11
391	hsa-miR-3940-3p	38	46	46	23	22	18
392	hsa-miR-3941	39	24	33	17	21	26
393	hsa-miR-3943	13670	16003	13701	14858	12258	9652
394	hsa-miR-3944-3p	32	32	32	28	24	26
395	hsa-miR-3945	38	44	55	22	25	32
396	hsa-miR-3960	168	139	169	165	110	113
397	hsa-miR-410-5p	964	1140	1046	771	913	683
398	hsa-miR-421	1456	1171	1391	626	462	628
399	hsa-miR-423-3p	2087	1814	2907	810	753	1315
400	hsa-miR-423-5p	1577	1292	1578	567	466	593
401	hsa-miR-424-3p	41	34	36	19	19	25
402	hsa-miR-424-5p	78	82	69	47	38	30

403	hsa-miR-425-5p	184	208	191	81	85	99
404	hsa-miR-4256	40	40	30	30	35	29
405	hsa-miR-4267	543	467	409	234	247	197
406	hsa-miR-4273	56	39	63	28	32	20
407	hsa-miR-4280	23	20	32	13	18	18
408	hsa-miR-4281	124	119	76	83	85	73
409	hsa-miR-4284	270	174	279	163	128	176
410	hsa-miR-4286	19	21	16	12	16	13
411	hsa-miR-4291	32	60	43	19	50	26
412	hsa-miR-4298	34	30	35	25	22	24
413	hsa-miR-4309	32	18	82	18	15	21
414	hsa-miR-4310	51	108	83	41	60	64
415	hsa-miR-4313	24	33	27	16	18	16
416	hsa-miR-4315	155	53	672	29	52	43
417	hsa-miR-4321	500	714	2172	536	1042	594
418	hsa-miR-4325	28	24	26	16	20	15
419	hsa-miR-4330	188	168	12932	85	172	110
420	hsa-miR-4417	26	21	27	17	19	23
421	hsa-miR-4420	45	36	47	30	33	35
422	hsa-miR-4423-5p	27	19	35	36	14	25
423	hsa-miR-4427	27	21	39	13	13	16
424	hsa-miR-4429	306	356	315	239	259	176
425	hsa-miR-4430	24	26	25	14	15	20
426	hsa-miR-4431	649	570	554	399	406	375
427	hsa-miR-4433-5p	72	166	138	62	168	61
428	hsa-miR-4443	4367	3261	2520	3333	1930	2096
429	hsa-miR-4444	18	25	25	13	26	21
430	hsa-miR-4448	1038	929	733	615	687	459
431	hsa-miR-4449	41	53	77	24	26	25
432	hsa-miR-4450	22	21	17	11	12	14
433	hsa-miR-4451	149	196	108	108	126	68
434	hsa-miR-4453	52	52	60	34	36	32
435	hsa-miR-4454	4141	3425	7268	2628	2567	2935
436	hsa-miR-4457	149	114	195	83	86	126
437	hsa-miR-4459	34	30	81	20	21	28
438	hsa-miR-4460	940	951	1041	606	624	540
439	hsa-miR-4461	39	46	46	20	26	23
440	hsa-miR-4465	52	87	75	35	60	45
441	hsa-miR-4466	33	35	41	28	24	26
442	hsa-miR-4467	27	28	36	22	21	23
443	hsa-miR-4468	663	499	547	312	370	508

444	hsa-miR-4473	52	39	39	33	29	30
445	hsa-miR-4475	26	22	28	18	19	32
446	hsa-miR-4479	24	35	37	16	19	20
447	hsa-miR-4482-5p	31	29	35	18	22	26
448	hsa-miR-4483	581	846	904	646	846	648
449	hsa-miR-4484	86	81	110	45	56	67
450	hsa-miR-4485	28	23	20	18	22	20
451	hsa-miR-4488	23	26	26	20	22	22
452	hsa-miR-4490	19	30	20	14	10	10
453	hsa-miR-4492	52	33	41	76	39	52
454	hsa-miR-4494	20	20	17	10	10	11
455	hsa-miR-4496	19	17	21	11	10	12
456	hsa-miR-4497	35	28	24	25	24	19
457	hsa-miR-449a	37	31	22	17	14	8
458	hsa-miR-449c-5p	99	71	46	32	24	15
459	hsa-miR-4502	2641	1907	1822	1973	2158	2008
460	hsa-miR-4504	19	18	16	11	18	12
461	hsa-miR-450a-5p	37	53	28	26	27	16
462	hsa-miR-450b-5p	260	322	188	153	146	94
463	hsa-miR-4515	81	68	67	77	53	56
464	hsa-miR-4517	474	553	431	401	490	377
465	hsa-miR-4518	23	27	34	12	22	19
466	hsa-miR-4520a-5p___hsa-miR-4520b-5p	32	26	269	20	27	31
467	hsa-miR-4521	49	45	60	50	37	43
468	hsa-miR-4527	34	25	32	18	24	20
469	hsa-miR-4530	33	24	94	17	17	21
470	hsa-miR-4532	36	37	43	25	24	24
471	hsa-miR-4535	724	454	515	443	433	462
472	hsa-miR-4537	19	16	18	10	11	10
473	hsa-miR-454-3p	253	245	263	166	119	136
474	hsa-miR-454-5p	113	78	99	40	34	46
475	hsa-miR-455-5p	46	39	41	24	17	18
476	hsa-miR-4634	64	110	83	66	82	67
477	hsa-miR-4635	16	18	20	12	12	14
478	hsa-miR-4638-3p	36	95	68	23	53	24
479	hsa-miR-4639-5p	17	16	22	11	11	12
480	hsa-miR-4642	19	26	33	16	18	19
481	hsa-miR-4644	24	20	22	13	11	16
482	hsa-miR-4648	69	166	123	44	152	72
483	hsa-miR-4650-3p	38	41	52	30	35	34

484	hsa-miR-4653-5p	227	374	304	172	292	196
485	hsa-miR-4655-3p	17	18	16	12	9	9
486	hsa-miR-4660	20	24	27	11	10	14
487	hsa-miR-4661-5p	30	22	29	12	15	15
488	hsa-miR-4662b	108	41	102	24	28	28
489	hsa-miR-4664-3p	20	18	26	10	12	12
490	hsa-miR-4665-3p	64	49	113	47	38	36
491	hsa-miR-4668-3p	15	15	16	9	9	8
492	hsa-miR-4668-5p	466	322	450	181	189	277
493	hsa-miR-4670-5p	56	50	44	40	48	38
494	hsa-miR-4673	46	38	60	24	21	32
495	hsa-miR-4676-3p	116	88	129	70	74	86
496	hsa-miR-4676-5p	19	27	20	13	17	12
497	hsa-miR-4677-3p	126	143	114	55	59	53
498	hsa-miR-4677-5p	74	62	85	52	50	94
499	hsa-miR-4680-5p	17	25	27	10	13	9
500	hsa-miR-4682	46	31	63	23	19	20
501	hsa-miR-4683	39	42	158	26	32	35
502	hsa-miR-4686	16	15	22	11	9	10
503	hsa-miR-4688	19	15	27	9	10	10
504	hsa-miR-4693-3p	40	56	63	32	28	32
505	hsa-miR-4695-3p	1266	3942	2566	1642	2202	1281
506	hsa-miR-4696	43	50	65	29	50	35
507	hsa-miR-4699-3p	137	154	109	121	119	83
508	hsa-miR-4699-5p	255	287	189	178	212	134
509	hsa-miR-4700-5p	98	72	140	36	46	36
510	hsa-miR-4701-3p	87	71	61	48	59	43
511	hsa-miR-4701-5p	22	42	31	22	26	31
512	hsa-miR-4707-3p	25	19	17	14	14	16
513	hsa-miR-4707-5p	15	18	18	15	11	18
514	hsa-miR-4708-3p	48	40	82	35	30	32
515	hsa-miR-4709-3p	21	18	23	9	13	15
516	hsa-miR-4711-3p	27	23	20	19	19	19
517	hsa-miR-4711-5p	20	22	19	13	17	18
518	hsa-miR-4712-3p	18	16	20	10	10	10
519	hsa-miR-4715-5p	19	15	33	11	12	13
520	hsa-miR-4717-3p	27	18	24	15	19	24
521	hsa-miR-4718	32	23	27	20	20	21
522	hsa-miR-4722-3p	59	71	71	60	58	47
523	hsa-miR-4722-5p	24	17	29	14	13	23
524	hsa-miR-4726-5p	48	39	62	19	23	30

525	hsa-miR-4729	36	19	25	21	19	21
526	hsa-miR-4732-5p	59	48	52	18	21	21
527	hsa-miR-4733-5p	47	44	41	18	20	23
528	hsa-miR-4734	48	29	43	34	27	28
529	hsa-miR-4735-5p	20	19	68	14	29	26
530	hsa-miR-4737	38	35	43	31	25	33
531	hsa-miR-4746-5p	96	97	100	55	56	54
532	hsa-miR-4747-3p	70	63	56	56	60	65
533	hsa-miR-4750-5p	17	23	22	14	14	15
534	hsa-miR-4755-3p	1596	489	2660	138	285	300
535	hsa-miR-4757-5p	54	29	31	27	17	19
536	hsa-miR-4758-3p	105	26	291	15	26	36
537	hsa-miR-4762-3p	31	27	33	17	26	19
538	hsa-miR-4763-3p	101	89	106	85	91	115
539	hsa-miR-4763-5p	36	28	35	21	22	29
540	hsa-miR-4764-3p	54	52	52	35	34	37
541	hsa-miR-4766-5p	113	120	101	88	83	85
542	hsa-miR-4767	19	30	23	17	20	17
543	hsa-miR-4770	206	212	184	175	149	108
544	hsa-miR-4772-3p	23	28	25	17	18	19
545	hsa-miR-4775	31	34	39	22	18	21
546	hsa-miR-4776-5p	548	721	639	303	421	336
547	hsa-miR-4778-5p	89	50	100	46	46	92
548	hsa-miR-4783-3p	20	17	18	13	12	13
549	hsa-miR-4786-5p	22	20	24	13	12	12
550	hsa-miR-4787-3p	47	94	62	32	83	34
551	hsa-miR-4787-5p	19	19	18	13	11	14
552	hsa-miR-4788	28	24	20	12	13	9
553	hsa-miR-4791	893	775	665	697	808	700
554	hsa-miR-4792	156	139	98	127	105	79
555	hsa-miR-4795-3p	37	29	36	22	21	22
556	hsa-miR-4798-5p	36	42	104	22	32	31
557	hsa-miR-4800-3p	26	48	59	17	24	24
558	hsa-miR-484	773	1033	882	412	421	422
559	hsa-miR-485-5p	766	524	545	522	513	605
560	hsa-miR-486-5p	264	292	263	149	152	150
561	hsa-miR-488-3p	16	23	18	6	11	8
562	hsa-miR-493-5p	28	38	45	64	77	71
563	hsa-miR-497-3p	39	26	38	15	15	32
564	hsa-miR-497-5p	29	28	32	15	12	14
565	hsa-miR-499a-5p	109	91	93	81	87	74

566	hsa-miR-499b-5p	125	144	128	121	105	79
567	hsa-miR-5000-3p	148	175	150	95	133	104
568	hsa-miR-5000-5p	22	17	22	14	12	10
569	hsa-miR-5001-3p	19	17	24	11	12	14
570	hsa-miR-5001-5p	28	30	29	27	21	21
571	hsa-miR-5002-5p	144	84	195	43	63	64
572	hsa-miR-5008-3p	26	24	27	16	14	19
573	hsa-miR-5009-3p	137	103	135	59	90	74
574	hsa-miR-500a-3p	594	447	436	232	197	277
575	hsa-miR-501-3p	122	102	74	50	45	60
576	hsa-miR-5010-5p	52	69	78	32	71	45
577	hsa-miR-503-5p	16	15	16	8	7	10
578	hsa-miR-505-3p	28	26	25	13	12	16
579	hsa-miR-5088-5p	16	15	49	6	10	11
580	hsa-miR-5089-3p	23	24	19	17	25	20
581	hsa-miR-5089-5p	88	71	67	65	63	89
582	hsa-miR-5091	22	26	19	12	10	16
583	hsa-miR-5093	19	22	22	11	14	13
584	hsa-miR-5094	15	16	16	9	8	13
585	hsa-miR-5096	31	35	31	20	21	22
586	hsa-miR-5100	102	162	121	93	104	70
587	hsa-miR-513c-3p	40	35	40	25	38	32
588	hsa-miR-516b-5p	84	93	119	66	50	70
589	hsa-miR-5190	28	22	31	10	12	11
590	hsa-miR-5191	18	17	22	10	10	10
591	hsa-miR-5192	32	36	30	25	23	18
592	hsa-miR-5195-3p	77	59	103	60	61	72
593	hsa-miR-5196-3p	559	958	1380	436	550	664
594	hsa-miR-5197-3p	31	28	31	14	23	18
595	hsa-miR-520d-3p	161	92	197	64	95	89
596	hsa-miR-532-5p	279	215	224	114	126	104
597	hsa-miR-539-3p	1558	1029	907	1068	572	563
598	hsa-miR-541-3p	86	111	103	42	41	46
599	hsa-miR-542-3p	195	146	208	71	81	82
600	hsa-miR-548ag	47	50	42	19	17	20
601	hsa-miR-548an	30	21	42	13	22	17
602	hsa-miR-548aq-5p	24	25	18	19	17	12
603	hsa-miR-548aw	44	37	47	27	27	40
604	hsa-miR-548b-3p	28	18	24	15	9	9
605	hsa-miR-548b-5p	215	182	171	84	62	65
606	hsa-miR-548ba	41	48	46	25	22	23

607	hsa-miR-548e-3p	126	115	101	69	65	57
608	hsa-miR-548e-5p	28	34	30	15	12	12
609	hsa-miR-548f-3p	19	32	32	19	26	27
610	hsa-miR-548h-5p	53	45	45	23	16	20
611	hsa-miR-548k	323	304	274	149	105	112
612	hsa-miR-548m	132	72	121	55	56	64
613	hsa-miR-548o-3p	116	88	91	50	32	31
614	hsa-miR-548y	25	21	18	12	15	15
615	hsa-miR-550a-5p	100	86	81	41	30	46
616	hsa-miR-552-3p	28	28	30	18	15	19
617	hsa-miR-553	47	50	47	36	30	31
618	hsa-miR-5582-3p	16	22	27	10	11	10
619	hsa-miR-5585-3p	81	86	96	42	56	65
620	hsa-miR-5587-3p	23	24	22	17	12	18
621	hsa-miR-5589-5p	220	202	326	306	278	360
622	hsa-miR-559	1197	1013	1373	709	823	1166
623	hsa-miR-561-5p	576	528	395	219	161	132
624	hsa-miR-563	21	24	28	16	19	12
625	hsa-miR-566	94	96	100	66	76	105
626	hsa-miR-567	93	83	110	65	70	90
627	hsa-miR-5683	45	45	57	32	35	30
628	hsa-miR-5685	392	336	375	263	267	358
629	hsa-miR-5688	178	169	197	92	107	130
630	hsa-miR-5689	46	42	53	30	32	35
631	hsa-miR-5691	72	43	84	29	35	38
632	hsa-miR-5693	78	46	112	37	43	39
633	hsa-miR-5694	365	442	441	322	350	328
634	hsa-miR-5699-3p	197	217	161	129	121	74
635	hsa-miR-5699-5p	31	25	35	16	15	14
636	hsa-miR-5701	23	17	43	12	15	13
637	hsa-miR-5706	24	23	19	13	16	9
638	hsa-miR-5707	48	43	51	40	35	32
639	hsa-miR-5708	33	29	28	14	11	16
640	hsa-miR-571	22	18	26	11	14	13
641	hsa-miR-573	61	58	47	55	58	50
642	hsa-miR-576-3p	138	118	136	100	86	89
643	hsa-miR-576-5p	52	37	44	15	12	15
644	hsa-miR-577	2355	2168	2102	1066	952	1154
645	hsa-miR-5787	203	191	164	181	180	220
646	hsa-miR-579-3p	74	85	63	47	48	34
647	hsa-miR-580-3p	646	645	428	582	665	441

648	hsa-miR-580-5p	98	89	101	53	66	53
649	hsa-miR-582-3p	296	433	174	135	146	64
650	hsa-miR-582-5p	51	70	33	20	27	16
651	hsa-miR-584-5p	17	17	32	7	10	12
652	hsa-miR-586	21	16	20	12	11	12
653	hsa-miR-587	117	97	124	82	88	108
654	hsa-miR-589-3p	34	45	50	25	23	23
655	hsa-miR-589-5p	260	277	264	132	131	142
656	hsa-miR-590-3p	110	121	86	67	49	41
657	hsa-miR-596	32	30	77	27	21	18
658	hsa-miR-598-3p	116	93	94	57	51	40
659	hsa-miR-598-5p	108	78	89	73	56	70
660	hsa-miR-600	104	82	87	49	42	44
661	hsa-miR-601	27	28	28	15	26	23
662	hsa-miR-605-3p	446	436	365	380	401	312
663	hsa-miR-606	17	19	20	14	14	15
664	hsa-miR-6076	18	15	15	10	7	8
665	hsa-miR-6079	38	27	30	27	21	25
666	hsa-miR-6080	17707	23983	31583	16684	13966	15192
667	hsa-miR-6081	122	62	117	53	22	54
668	hsa-miR-6082	28	29	81	18	21	14
669	hsa-miR-6083	25	19	28	12	13	13
670	hsa-miR-6086	640	667	580	275	373	299
671	hsa-miR-6089	26	29	25	18	23	22
672	hsa-miR-609	35	24	38	13	16	10
673	hsa-miR-612	26	18	33	9	11	14
674	hsa-miR-6126	80	75	94	61	59	51
675	hsa-miR-6132	117	114	162	96	90	109
676	hsa-miR-6133	32	36	32	18	36	32
677	hsa-miR-615-3p	468	555	400	201	187	178
678	hsa-miR-615-5p	160	182	129	131	102	96
679	hsa-miR-618	37	47	44	20	28	22
680	hsa-miR-619-5p	42	33	43	18	22	29
681	hsa-miR-624-5p	91	83	110	68	70	69
682	hsa-miR-625-3p	19	20	19	10	10	11
683	hsa-miR-625-5p	75	55	46	35	25	35
684	hsa-miR-628-5p	61	72	57	29	21	22
685	hsa-miR-629-5p	132	121	122	69	58	89
686	hsa-miR-630	19	18	22	12	11	15
687	hsa-miR-631	33	20	26	21	21	21
688	hsa-miR-637	20	19	20	11	11	10

689	hsa-miR-638	254	256	217	207	174	146
690	hsa-miR-641	274	236	211	90	75	73
691	hsa-miR-643	637	682	514	504	486	382
692	hsa-miR-644a	288	345	346	308	281	296
693	hsa-miR-6499-3p	43	31	55	30	24	43
694	hsa-miR-6499-5p	18	15	19	10	12	13
695	hsa-miR-650	86	88	88	48	53	55
696	hsa-miR-6501-3p	25	31	30	19	26	19
697	hsa-miR-6502-5p	19	18	27	9	15	11
698	hsa-miR-6503-3p	16	18	17	11	11	13
699	hsa-miR-6503-5p	189	288	233	194	211	148
700	hsa-miR-6506-5p	35	35	29	39	38	28
701	hsa-miR-651-5p	27	24	31	64	97	104
702	hsa-miR-6510-3p	15	15	19	10	9	12
703	hsa-miR-6512-3p	38	29	58	17	24	21
704	hsa-miR-6513-3p	64	81	65	81	67	64
705	hsa-miR-652-3p	56	62	42	29	31	34
706	hsa-miR-660-5p	226	199	172	139	103	89
707	hsa-miR-662	595	1025	877	448	560	552
708	hsa-miR-663a	35	36	48	44	38	44
709	hsa-miR-663b	30	28	42	22	22	21
710	hsa-miR-664b-5p	66	64	43	40	43	32
711	hsa-miR-665	31	25	35	16	19	21
712	hsa-miR-668-3p	154	69	223	84	67	114
713	hsa-miR-671-3p	95	88	108	44	53	49
714	hsa-miR-671-5p	56	77	66	19	16	18
715	hsa-miR-6717-5p	430	368	602	162	198	238
716	hsa-miR-6719-3p	664	762	485	584	577	373
717	hsa-miR-6723-5p	34	28	23	16	14	19
718	hsa-miR-6724-5p	59	72	67	38	37	41
719	hsa-miR-6726-3p	30	40	22	24	28	19
720	hsa-miR-6728-5p	15	18	62	12	9	11
721	hsa-miR-6729-5p	21	17	16	13	14	14
722	hsa-miR-6730-3p	752	902	657	522	563	530
723	hsa-miR-6731-3p	55	61	49	42	43	33
724	hsa-miR-6735-3p	2400	1683	1275	1131	659	594
725	hsa-miR-6735-5p	22	16	16	9	9	11
726	hsa-miR-6737-5p	18	15	17	12	11	12
727	hsa-miR-6739-5p	24	35	31	23	20	24
728	hsa-miR-6743-5p	22	20	27	16	13	15
729	hsa-miR-6746-3p	112	126	112	79	88	64

730	hsa-miR-6749-5p	37	29	36	16	13	17
731	hsa-miR-675-5p	54	44	130	32	39	36
732	hsa-miR-6755-3p	36	19	40	17	15	16
733	hsa-miR-6757-5p	1741	281	4266	163	441	472
734	hsa-miR-676-3p	25	30	27	13	13	17
735	hsa-miR-6761-5p	47	42	24	31	27	25
736	hsa-miR-6763-3p	24	29	33	20	14	13
737	hsa-miR-6765-3p	988	1382	1006	770	792	764
738	hsa-miR-6768-5p	16	20	21	10	9	11
739	hsa-miR-6769a-3p	47	35	91	13	14	13
740	hsa-miR-6770-3p	19	15	22	11	8	12
741	hsa-miR-6772-5p	214	182	244	132	142	171
742	hsa-miR-6773-5p	18	18	19	10	12	10
743	hsa-miR-6776-3p	28	21	37	15	26	21
744	hsa-miR-6780a-3p	79	69	87	50	31	51
745	hsa-miR-6783-3p	26	22	46	10	12	14
746	hsa-miR-6784-3p	37	29	66	16	29	25
747	hsa-miR-6784-5p	27	22	31	21	16	22
748	hsa-miR-6785-3p	126	191	172	83	82	101
749	hsa-miR-6785-5p	39	22	54	10	14	15
750	hsa-miR-6786-3p	34	19	30	14	16	16
751	hsa-miR-6787-5p	65	57	41	38	40	33
752	hsa-miR-6788-5p	38	19	25	20	17	22
753	hsa-miR-6789-5p	560	792	602	530	574	510
754	hsa-miR-6792-5p	65	53	81	44	52	66
755	hsa-miR-6797-5p	26	24	44	16	15	19
756	hsa-miR-6805-3p	21	23	34	10	12	17
757	hsa-miR-6806-5p	27	28	30	19	21	35
758	hsa-miR-6809-5p	19	18	21	12	12	11
759	hsa-miR-6815-5p	24	26	23	19	14	15
760	hsa-miR-6816-5p	549	832	547	341	475	321
761	hsa-miR-6819-5p	23	18	25	18	10	13
762	hsa-miR-6821-5p	19	20	34	13	16	13
763	hsa-miR-6822-3p	15	17	28	10	16	11
764	hsa-miR-6822-5p	70	143	95	75	80	79
765	hsa-miR-6824-5p	23	19	19	13	12	15
766	hsa-miR-6825-3p	20	23	28	13	15	14
767	hsa-miR-6826-3p	53	81	45	39	62	31
768	hsa-miR-6826-5p	400	497	441	227	241	254
769	hsa-miR-6832-3p	208	157	317	83	130	122
770	hsa-miR-6836-3p	27	45	43	22	34	36

771	hsa-miR-6837-3p	17	22	22	10	10	9
772	hsa-miR-6839-5p	63	67	196	34	47	33
773	hsa-miR-6840-5p	37	45	53	28	29	36
774	hsa-miR-6842-3p	22	27	30	15	17	16
775	hsa-miR-6844	64	64	80	41	42	67
776	hsa-miR-6852-3p	56	29	57	36	18	26
777	hsa-miR-6852-5p	25	16	28	8	10	9
778	hsa-miR-6853-3p	93	43	136	18	31	30
779	hsa-miR-6854-5p	50	49	65	30	30	34
780	hsa-miR-6855-5p	98	66	99	44	51	76
781	hsa-miR-6856-5p	26	22	23	14	17	21
782	hsa-miR-6859-3p	15	16	17	9	10	12
783	hsa-miR-6859-5p	38	29	152	13	19	18
784	hsa-miR-6868-5p	35	39	35	19	22	23
785	hsa-miR-6869-3p	34	26	35	31	18	24
786	hsa-miR-6869-5p	104	103	124	156	100	113
787	hsa-miR-6872-3p	92	110	111	62	68	91
788	hsa-miR-6876-3p	23	24	50	19	20	31
789	hsa-miR-6877-3p	17	25	20	17	20	15
790	hsa-miR-6878-5p	168	163	206	109	144	147
791	hsa-miR-6881-3p	35	15	27	31	4	14
792	hsa-miR-6892-5p	30	30	40	22	19	25
793	hsa-miR-6894-5p	34	34	47	17	21	27
794	hsa-miR-7-1-3p	24	28	21	13	12	12
795	hsa-miR-7-2-3p	41	44	39	26	28	20
796	hsa-miR-7-5p	19	18	61	5	4	5
797	hsa-miR-708-3p	53	49	52	41	26	22
798	hsa-miR-708-5p	42	42	38	25	24	25
799	hsa-miR-711	62	95	48	53	53	74
800	hsa-miR-7151-3p	30	33	40	15	17	25
801	hsa-miR-7153-5p	26	26	27	21	18	13
802	hsa-miR-7156-5p	29	32	34	20	23	19
803	hsa-miR-7159-5p	52	55	61	32	39	32
804	hsa-miR-7160-5p	20	18	19	13	10	13
805	hsa-miR-7161-3p	259	265	952	157	255	164
806	hsa-miR-7161-5p	28	20	32	40	27	20
807	hsa-miR-718	196	137	164	132	115	94
808	hsa-miR-744-3p	18	16	17	7	8	8
809	hsa-miR-744-5p	637	725	600	343	291	292
810	hsa-miR-7515	72	57	161	48	48	50
811	hsa-miR-761	31	45	142	19	32	33

812	hsa-miR-762	471	519	444	391	356	329
813	hsa-miR-7641	64	31	380	16	18	21
814	hsa-miR-767-5p	47	34	134	19	23	18
815	hsa-miR-769-3p	21	21	23	10	11	13
816	hsa-miR-769-5p	574	641	644	150	130	168
817	hsa-miR-7703	2951	2671	2368	2111	1941	2004
818	hsa-miR-7704	97	109	115	83	82	81
819	hsa-miR-7705	540	556	483	432	387	371
820	hsa-miR-7706	101	104	164	53	96	94
821	hsa-miR-7844-5p	22	16	20	9	12	15
822	hsa-miR-7846-3p	45	33	33	29	26	26
823	hsa-miR-7847-3p	22	20	25	13	10	12
824	hsa-miR-7974	1162	742	818	775	613	635
825	hsa-miR-7977	487	604	503	317	412	466
826	hsa-miR-8054	34	36	30	23	18	14
827	hsa-miR-8058	366	391	302	290	314	257
828	hsa-miR-8059	20	18	24	14	12	12
829	hsa-miR-8061	46	62	61	30	58	32
830	hsa-miR-8062	20	20	18	12	16	14
831	hsa-miR-8063	21	23	32	13	18	15
832	hsa-miR-8065	24	16	28	12	22	12
833	hsa-miR-8069	52	83	85	50	74	52
834	hsa-miR-8072	40	39	41	28	28	38
835	hsa-miR-8073	16	17	21	13	13	15
836	hsa-miR-8074	32	30	37	17	24	22
837	hsa-miR-8075	34	34	102	25	30	22
838	hsa-miR-8076	24	22	21	13	13	12
839	hsa-miR-8078	19	20	16	18	14	16
840	hsa-miR-8082	136	134	134	120	141	115
841	hsa-miR-8083	20	24	21	10	11	12
842	hsa-miR-8088	16	21	20	8	11	11
843	hsa-miR-873-3p	95	116	87	68	65	60
844	hsa-miR-874-3p	145	199	119	77	73	71
845	hsa-miR-875-3p	25	28	19	15	15	17
846	hsa-miR-877-5p	61	58	49	21	20	20
847	hsa-miR-885-3p	54	63	71	28	38	38
848	hsa-miR-887-3p	24	33	24	11	11	13
849	hsa-miR-887-5p	49	49	52	24	24	25
850	hsa-miR-889-5p	22	21	29	17	14	16
851	hsa-miR-891a-3p	16	17	22	8	8	7
852	hsa-miR-891a-5p	54	36	51	21	21	29

853	hsa-miR-892a	24	21	18	18	15	9
854	hsa-miR-892c-3p	78	88	63	62	68	39
855	hsa-miR-9-3p	127	134	169	69	78	72
856	hsa-miR-9-5p	524	674	520	201	206	173
857	hsa-miR-921	15	18	19	8	9	7
858	hsa-miR-922	40	34	33	20	27	23
859	hsa-miR-92a-1-5p	79	37	70	13	12	21
860	hsa-miR-92a-3p	38489	22715	42878	20666	12476	21160
861	hsa-miR-92b-3p	2405	2625	2576	1113	901	917
862	hsa-miR-93-3p	63	45	81	27	30	41
863	hsa-miR-93-5p	2800	2635	2972	1355	1427	1467
864	hsa-miR-937-3p	102	91	142	65	88	76
865	hsa-miR-938	877	856	1028	419	493	551
866	hsa-miR-939-5p	18	17	21	13	15	11
867	hsa-miR-940	21	34	26	13	15	13
868	hsa-miR-941	1016	855	1139	338	316	462
869	hsa-miR-942-3p	66	44	72	32	36	37
870	hsa-miR-942-5p	89	62	65	31	26	32
871	hsa-miR-96-5p	234	237	175	128	100	128
872	hsa-miR-98-5p	118	128	228	95	87	88
873	hsa-miR-99a-3p	25	26	34	17	22	15
874	hsa-miR-99a-5p	341	233	276	120	82	139
875	hsa-miR-99b-3p	22	23	20	10	14	12
876	hsa-miR-99b-5p	650	645	504	253	294	312
