# Supplementary material for: N6-Adenosine Methylation in MiRNAs
Source: PLoS One. 2015 Feb 27;10(2):e0118438. doi: 10.1371/journal.pone.0118438 (PMC4344304; doi:10.1371/journal.pone.0118438)
Supplement: S2 Table — We detected 249 miRNAs with significantly increased levels (>1.5-fold change) (a) and 35 miRNAs with significantly decreased levels (b). Means and standard deviations of three compared pairs of scrambled and FTO specific siRNA treated cells are given. (PDF) [file pone.0118438.s002.pdf]

Supplementary Table 2.

List of deregulated mature miRNAs in *FTO* knockdown cells. We detected 249 miRNAs with significantly increased levels (>1.5-fold change) (a) and 35 miRNAs with significantly decreased levels (b). Means and standard deviations of three compared pairs of scrambled and FTO specific siRNA treated cells are given.

a. Upregulated miRNAs in *FTO* deficient cells.

	Feature	**Mean**	**SD**
1	hsa-miR-6505-5p	9.83	4.61
2	hsa-miR-651-5p	6.49	1.61
3	hsa-miR-493-5p	3.99	0.98
4	hsa-miR-6739-3p	3.84	1.03
5	hsa-miR-221-3p	2.79	0.40
6	hsa-miR-4492	2.62	0.43
7	hsa-miR-5589-5p	2.62	0.51
8	hsa-miR-1245b-5p	2.53	0.51
9	hsa-miR-6763-5p	2.42	0.27
10	hsa-miR-1246	2.41	0.49
11	hsa-miR-23c	2.37	0.23
12	hsa-miR-4489	2.34	0.97
13	hsa-let-7b-5p	2.31	0.17
14	hsa-miR-6869-5p	2.30	0.80
15	hsa-miR-7161-5p	2.29	0.98
16	hsa-miR-3681-5p	2.28	0.36
17	hsa-miR-1324	2.27	0.09
18	hsa-miR-663a	2.18	0.50
19	hsa-miR-30a-3p	2.17	0.15
20	hsa-miR-6795-3p	2.16	0.67
21	hsa-miR-4664-5p	2.15	0.69
22	hsa-miR-6506-5p	2.13	0.32
23	hsa-miR-1258	2.11	0.44
24	hsa-miR-5787	2.11	0.32
25	hsa-miR-6758-5p	2.11	0.23
26	hsa-miR-127-5p	2.10	0.67
27	hsa-miR-5682	2.09	0.20
28	hsa-miR-6513-3p	2.07	0.54
29	hsa-miR-1257	2.04	0.25
30	hsa-miR-646	2.03	0.68
31	hsa-miR-635	2.02	0.44
32	hsa-miR-302a-5p	2.02	0.03
33	hsa-miR-6090	2.02	0.47

34	hsa-miR-3616-3p	2.01	0.43
35	hsa-miR-4502	1.99	0.38
36	hsa-miR-580-3p	1.99	0.13
37	hsa-miR-573	1.98	0.06
38	hsa-miR-4763-3p	1.98	0.18
39	hsa-miR-144-3p	1.97	0.43
40	hsa-miR-3609	1.97	0.24
41	hsa-miR-5088-3p	1.97	0.70
42	hsa-miR-5089-5p	1.96	0.45
43	hsa-miR-924	1.95	0.38
44	hsa-miR-4747-3p	1.94	0.22
45	hsa-miR-6088	1.93	0.45
46	hsa-miR-152-3p	1.93	0.16
47	hsa-miR-200b-5p	1.93	0.35
48	hsa-miR-4791	1.92	0.25
49	hsa-miR-711	1.92	0.83
50	hsa-miR-4483	1.92	0.54
51	hsa-miR-1182	1.92	0.73
52	hsa-miR-4423-5p	1.92	0.81
53	hsa-miR-1275	1.92	0.41
54	hsa-let-7f-2-3p	1.89	0.52
55	hsa-miR-8082	1.88	0.30
56	hsa-miR-5089-3p	1.88	0.26
57	hsa-miR-1203	1.87	0.60
58	hsa-miR-6791-5p	1.87	0.78
59	hsa-miR-668-5p	1.86	0.70
60	hsa-miR-644a	1.85	0.38
61	hsa-miR-506-3p	1.85	0.23
62	hsa-miR-30e-3p	1.85	0.04
63	hsa-miR-382-5p	1.85	0.80
64	hsa-miR-3607-5p	1.84	0.43
65	hsa-miR-485-5p	1.84	0.34
66	hsa-miR-4475	1.84	0.32
67	hsa-miR-4731-5p	1.82	0.82
68	hsa-miR-3617-3p	1.82	0.47
69	hsa-miR-3920	1.82	0.20
70	hsa-miR-1238-5p	1.81	0.56
71	hsa-miR-656-5p	1.80	0.76
72	hsa-miR-548f-3p	1.77	0.30
73	hsa-miR-4485	1.77	0.30
74	hsa-miR-605-3p	1.77	0.17
75	hsa-miR-8078	1.76	0.29
76	hsa-miR-3195	1.76	0.38
77	hsa-miR-6806-5p	1.76	0.35

78	hsa-miR-4517	1.75	0.12
79	hsa-miR-4444	1.75	0.34
80	hsa-miR-3680-3p	1.75	0.40
81	hsa-miR-4677-5p	1.75	0.28
82	hsa-miR-4634	1.74	0.40
83	hsa-miR-19a-5p	1.74	0.22
84	hsa-miR-4256	1.74	0.13
85	hsa-miR-3943	1.74	0.53
86	hsa-miR-4701-5p	1.73	0.40
87	hsa-miR-4521	1.73	0.43
88	hsa-miR-4707-5p	1.73	0.41
89	hsa-miR-6759-5p	1.73	0.61
90	hsa-miR-5579-3p	1.72	0.30
91	hsa-miR-4670-5p	1.72	0.27
92	hsa-miR-5703	1.72	0.63
93	hsa-miR-1266-3p	1.72	0.23
94	hsa-miR-4515	1.72	0.26
95	hsa-miR-4717-3p	1.72	0.52
96	hsa-miR-6125	1.72	0.45
97	hsa-miR-6877-3p	1.72	0.40
98	hsa-miR-6133	1.71	0.46
99	hsa-miR-363-5p	1.71	0.27
100	hsa-miR-4488	1.71	0.14
101	hsa-miR-1261	1.70	0.10
102	hsa-miR-145-3p	1.70	0.20
103	hsa-miR-5195-3p	1.70	0.43
104	hsa-miR-4722-3p	1.70	0.46
105	hsa-miR-1181	1.70	0.57
106	hsa-miR-1237-5p	1.70	0.35
107	hsa-miR-3622b-3p	1.70	0.14
108	hsa-miR-6789-5p	1.70	0.28
109	hsa-let-7i-5p	1.70	0.35
110	hsa-miR-337-3p	1.69	0.37
111	hsa-miR-6780b-5p	1.69	0.34
112	hsa-miR-566	1.69	0.23
113	hsa-miR-499a-5p	1.68	0.28
114	hsa-miR-3940-5p	1.68	0.21
115	hsa-miR-3192-3p	1.68	0.61
116	hsa-miR-513c-3p	1.68	0.48
117	hsa-miR-4711-3p	1.68	0.14
118	hsa-miR-8069	1.68	0.48
119	hsa-miR-631	1.67	0.45
120	hsa-miR-3942-5p	1.67	0.31
121	hsa-miR-3150b-3p	1.67	0.17

122	hsa-miR-3928-5p	1.67	0.02
123	hsa-miR-6792-5p	1.66	0.34
124	hsa-miR-1263	1.66	0.19
125	hsa-miR-587	1.66	0.20
126	hsa-miR-1272	1.66	0.05
127	hsa-miR-6822-5p	1.66	0.57
128	hsa-miR-3960	1.66	0.43
129	hsa-miR-1250-5p	1.66	0.20
130	hsa-miR-8066	1.65	0.39
131	hsa-miR-298	1.65	0.20
132	hsa-miR-136-5p	1.65	0.32
133	hsa-miR-4535	1.65	0.34
134	hsa-miR-30b-3p	1.65	0.32
135	hsa-miR-8058	1.64	0.06
136	hsa-miR-3934-5p	1.64	0.39
137	hsa-miR-6722-3p	1.64	0.28
138	hsa-miR-6836-3p	1.64	0.11
139	hsa-miR-3652	1.63	0.40
140	hsa-miR-653-3p	1.63	0.29
141	hsa-miR-3682-3p	1.63	0.20
142	hsa-miR-5694	1.63	0.26
143	hsa-miR-3944-3p	1.63	0.17
144	hsa-miR-4999-5p	1.63	0.24
145	hsa-miR-431-3p	1.63	0.42
146	hsa-miR-6503-5p	1.63	0.52
147	hsa-miR-4745-3p	1.62	0.38
148	hsa-miR-4699-3p	1.62	0.24
149	hsa-miR-3662	1.62	0.05
150	hsa-miR-4652-5p	1.62	0.45
151	hsa-miR-26b-5p	1.62	0.32
152	hsa-miR-5001-5p	1.62	0.39
153	hsa-miR-6719-3p	1.62	0.23
154	hsa-miR-29b-2-5p	1.62	0.34
155	hsa-miR-4417	1.61	0.25
156	hsa-miR-5685	1.61	0.17
157	hsa-miR-521	1.61	0.50
158	hsa-miR-137	1.61	0.27
159	hsa-miR-1908-3p	1.61	0.36
160	hsa-miR-6863	1.61	0.23
161	hsa-miR-6827-5p	1.60	0.41
162	hsa-miR-3614-5p	1.60	0.16
163	hsa-miR-3182	1.60	0.10
164	hsa-miR-4729	1.60	0.39
165	hsa-miR-6726-3p	1.60	0.14

166	hsa-miR-508-5p	1.60	0.35
167	hsa-miR-4504	1.59	0.40
168	hsa-miR-4792	1.59	0.13
169	hsa-miR-3163	1.59	0.36
170	hsa-miR-4667-5p	1.59	0.34
171	hsa-miR-1184	1.59	0.52
172	hsa-miR-6823-5p	1.58	0.29
173	hsa-miR-197-5p	1.58	0.28
174	hsa-miR-6793-5p	1.58	0.18
175	hsa-miR-98-3p	1.58	0.10
176	hsa-miR-4747-5p	1.58	0.40
177	hsa-miR-567	1.58	0.14
178	hsa-miR-6089	1.57	0.07
179	hsa-miR-4778-5p	1.57	0.41
180	hsa-miR-499b-5p	1.57	0.47
181	hsa-miR-1256	1.57	0.14
182	hsa-miR-4711-5p	1.57	0.16
183	hsa-miR-6739-5p	1.57	0.47
184	hsa-miR-8072	1.57	0.13
185	hsa-miR-7704	1.56	0.26
186	hsa-miR-4497	1.56	0.16
187	hsa-miR-6761-5p	1.56	0.32
188	hsa-miR-4737	1.56	0.17
189	hsa-miR-2115-3p	1.55	0.12
190	hsa-miR-1910-3p	1.55	0.13
191	hsa-miR-4281	1.55	0.18
192	hsa-miR-601	1.55	0.40
193	hsa-miR-606	1.55	0.18
194	hsa-miR-3131	1.55	0.41
195	hsa-miR-4734	1.55	0.35
196	hsa-miR-4766-5p	1.55	0.11
197	hsa-miR-4420	1.55	0.27
198	hsa-miR-3196	1.55	0.26
199	hsa-miR-4718	1.55	0.24
200	hsa-miR-4524a-3p	1.55	0.37
201	hsa-miR-4767	1.55	0.33
202	hsa-miR-4650-3p	1.55	0.32
203	hsa-miR-4739	1.55	0.34
204	hsa-miR-4503	1.55	0.11
205	hsa-miR-6132	1.55	0.27
206	hsa-miR-6845-3p	1.54	0.35
207	hsa-miR-6079	1.54	0.06
208	hsa-miR-8073	1.54	0.21
209	hsa-miR-1289	1.54	0.28

210	hsa-miR-3137	1.54	0.09
211	hsa-miR-6816-3p	1.54	0.36
212	hsa-miR-203b-3p	1.54	0.22
213	hsa-miR-4292	1.54	0.43
214	hsa-miR-6729-5p	1.54	0.20
215	hsa-miR-6499-3p	1.54	0.10
216	hsa-miR-6869-3p	1.54	0.35
217	hsa-miR-5707	1.53	0.34
218	hsa-miR-7703	1.53	0.03
219	hsa-miR-541-5p	1.53	0.45
220	hsa-miR-6790-3p	1.53	0.06
221	hsa-miR-200c-3p	1.52	0.22
222	hsa-miR-410-5p	1.52	0.28
223	hsa-miR-7705	1.52	0.16
224	hsa-miR-5004-3p	1.52	0.29
225	hsa-miR-7974	1.52	0.17
226	hsa-miR-3651	1.52	0.30
227	hsa-miR-6788-5p	1.52	0.38
228	hsa-miR-6515-5p	1.52	0.34
229	hsa-miR-372-3p	1.52	0.18
230	hsa-miR-6876-3p	1.52	0.35
231	hsa-miR-1343-5p	1.52	0.36
232	hsa-miR-1231	1.52	0.22
233	hsa-miR-4524b-3p	1.52	0.49
234	hsa-miR-762	1.52	0.22
235	hsa-miR-3692-3p	1.52	0.15
236	hsa-miR-3923	1.52	0.14
237	hsa-miR-33b-3p	1.52	0.33
238	hsa-miR-3145-3p	1.51	0.35
239	hsa-miR-643	1.51	0.16
240	hsa-miR-3620-5p	1.51	0.33
241	hsa-miR-6878-5p	1.51	0.28
242	hsa-miR-559	1.51	0.22
243	hsa-miR-3613-5p	1.50	0.19
244	hsa-miR-4467	1.50	0.30
245	hsa-miR-7977	1.50	0.18
246	hsa-miR-7846-3p	1.50	0.13
247	hsa-miR-6501-3p	1.50	0.32
248	hsa-miR-4533	1.50	0.30
249	hsa-miR-624-5p	1.50	0.32

b. Downregulated miRNAs in *FTO* deficient cells.

	Feature	**Mean**	**SD**
1	hsa-miR-22-3p	0.28	0.02
2	hsa-miR-196a-5p	0.35	0.06
3	hsa-miR-7-5p	0.40	0.23
4	hsa-miR-769-5p	0.49	0.07
5	hsa-miR-92a-1-5p	0.52	0.17
6	hsa-miR-671-5p	0.55	0.15
7	hsa-miR-6769a-3p	0.56	0.28
8	hsa-miR-27b-3p	0.56	0.07
9	hsa-miR-215-5p	0.61	0.07
10	hsa-miR-1180-3p	0.63	0.07
11	hsa-miR-576-5p	0.63	0.02
12	hsa-miR-28-3p	0.65	0.03
13	hsa-miR-25-3p	0.66	0.04
14	hsa-miR-449c-5p	0.66	0.05
15	hsa-miR-641	0.67	0.03
16	hsa-miR-330-3p	0.67	0.06
17	hsa-miR-1286	0.68	0.07
18	hsa-miR-561-5p	0.69	0.11
19	hsa-miR-9-5p	0.69	0.11
20	hsa-miR-218-5p	0.69	0.12
21	hsa-miR-195-5p	0.70	0.11
22	hsa-miR-148a-5p	0.71	0.10
23	hsa-miR-16-5p	0.71	0.04
24	hsa-miR-31-5p	0.71	0.08
25	hsa-miR-1910-5p	0.72	0.17
26	hsa-miR-3176	0.72	0.12
27	hsa-miR-4661-3p	0.73	0.19
28	hsa-miR-877-5p	0.73	0.01
29	hsa-miR-148b-5p	0.73	0.10
30	hsa-miR-423-5p	0.74	0.04
31	hsa-miR-941	0.74	0.03
32	hsa-miR-181b-5p	0.74	0.11
33	hsa-miR-548b-5p	0.75	0.08
34	hsa-miR-30e-5p	0.75	0.19
35	hsa-miR-21-5p	0.76	0.18
