# Supplementary material for: N6-Adenosine Methylation in MiRNAs
Source: PLoS One. 2015 Feb 27;10(2):e0118438. doi: 10.1371/journal.pone.0118438 (PMC4344304; doi:10.1371/journal.pone.0118438)
Supplement: S3 Table — Means of enrichment compared to IgG samples and their standard deviations are shown. (PDF) [file pone.0118438.s003.pdf]

Supplementary Table 3. List of miRNAs significantly enriched (>2-fold change) in RNA samples immunoprecipitated with anti-me6A antibody compared to those captured by anti-IgG antibody (n=239). Means of enrichment compared to IgG samples and their standard deviations are shown.

	feature	**mean**	**SD**
1	hsa-miR-500a-3p	485.21	464.98
2	hsa-miR-103a-3p	431.65	260.54
3	hsa-miR-136-5p	262.50	202.93
4	hsa-miR-378a-3p	240.31	180.44
5	hsa-miR-1910-3p	217.40	77.61
6	hsa-miR-421	205.75	107.11
7	hsa-miR-28-5p	185.08	160.06
8	hsa-miR-3681-5p	179.17	54.76
9	hsa-miR-374b-5p	146.77	97.05
10	hsa-miR-6806-5p	133.11	32.84
11	hsa-miR-146b-5p	125.42	62.17
12	hsa-miR-320a	120.06	11.23
13	hsa-miR-548e-3p	116.69	68.97
14	hsa-miR-1255a	107.96	90.27
15	hsa-miR-4653-5p	107.21	9.94
16	hsa-miR-4298	102.29	33.42
17	hsa-miR-4791	100.95	47.98
18	hsa-miR-215-5p	99.50	73.15
19	hsa-miR-4502	98.28	25.63
20	hsa-miR-485-5p	85.04	15.14
21	hsa-miR-580-3p	79.82	22.63
22	hsa-miR-10a-5p	78.13	45.89
23	hsa-miR-1246	77.06	43.85
24	hsa-miR-3672	75.69	72.69
25	hsa-miR-3613-3p	71.96	40.91
26	hsa-miR-4535	69.55	25.04
27	hsa-miR-191-5p	62.58	11.94
28	hsa-miR-10b-5p	61.22	21.15
29	hsa-miR-1322	58.82	24.90
30	hsa-miR-7703	55.20	11.13
31	hsa-miR-4648	52.81	22.87
32	hsa-miR-3688-5p	50.45	44.18
33	hsa-miR-183-5p	50.18	30.53
34	hsa-miR-7153-5p	50.14	19.35
35	hsa-miR-559	50.09	15.98

36	hsa-miR-377-3p	49.57	18.77
37	hsa-let-7f-5p	49.18	10.12
38	hsa-miR-3662	48.97	29.93
39	hsa-miR-628-5p	48.36	15.88
40	hsa-miR-200b-3p	48.06	35.28
41	hsa-miR-31-5p	46.62	27.05
42	hsa-miR-1261	45.62	12.76
43	hsa-miR-1914-5p	44.73	26.93
44	hsa-miR-223-3p	43.20	23.00
45	hsa-miR-3176	42.97	23.68
46	hsa-miR-199b-5p	42.71	32.42
47	hsa-miR-612	41.48	24.97
48	hsa-miR-4668-5p	40.85	10.11
49	hsa-miR-4527	40.62	21.86
50	hsa-miR-888-5p	40.24	37.99
51	hsa-miR-3128	37.03	18.24
52	hsa-miR-22-3p	35.74	3.76
53	hsa-miR-1468-3p	35.41	10.74
54	hsa-miR-4524a-3p	34.40	6.91
55	hsa-miR-151a-3p	34.39	24.73
56	hsa-miR-130b-3p	33.92	14.70
57	hsa-let-7g-5p	33.36	16.17
58	hsa-miR-5688	31.50	1.40
59	hsa-miR-4468	30.80	20.43
60	hsa-miR-539-3p	30.57	18.32
61	hsa-miR-222-3p	30.29	15.51
62	hsa-miR-877-5p	29.87	14.31
63	hsa-miR-423-3p	29.64	13.29
64	hsa-miR-605-3p	28.77	7.50
65	hsa-miR-4778-5p	28.75	9.53
66	hsa-miR-708-5p	28.38	16.10
67	hsa-miR-501-3p	28.05	7.12
68	hsa-miR-16-5p	27.63	17.75
69	hsa-miR-6730-3p	26.63	8.86
70	hsa-miR-3616-3p	25.98	19.64
71	hsa-miR-4429	25.87	13.99
72	hsa-miR-567	24.65	19.31
73	hsa-miR-6788-5p	23.81	14.53
74	hsa-let-7a-5p	23.77	6.71
75	hsa-miR-181a-5p	22.24	12.79
76	hsa-miR-363-3p	21.72	16.88

77	hsa-miR-7974	21.29	6.33
78	hsa-miR-4517	21.13	1.72
79	hsa-let-7e-5p	20.74	4.93
80	hsa-miR-5190	19.48	7.43
81	hsa-miR-340-5p	18.49	14.42
82	hsa-miR-942-3p	18.47	5.35
83	hsa-miR-577	18.09	4.84
84	hsa-miR-3175	17.81	6.36
85	hsa-miR-4711-5p	17.68	6.61
86	hsa-miR-5683	17.39	3.69
87	hsa-miR-181b-5p	17.31	9.31
88	hsa-miR-671-5p	16.00	8.37
89	hsa-miR-1273e	15.80	10.21
90	hsa-miR-7151-3p	15.65	1.90
91	hsa-miR-26a-5p	15.38	0.89
92	hsa-miR-4530	14.81	6.46
93	hsa-miR-152-3p	14.29	2.34
94	hsa-miR-4454	14.09	4.33
95	hsa-miR-3173-3p	13.69	7.87
96	hsa-miR-423-5p	13.44	3.87
97	hsa-miR-519e-5p	13.43	7.56
98	hsa-miR-765	13.42	3.96
99	hsa-miR-3605-5p	13.00	1.64
100	hsa-miR-1258	12.96	1.40
101	hsa-miR-641	12.90	6.17
102	hsa-miR-4755-3p	12.87	7.78
103	hsa-miR-431-5p	12.86	5.49
104	hsa-miR-148a-5p	12.85	10.30
105	hsa-miR-600	12.80	5.91
106	hsa-miR-125b-2-3p	12.78	9.07
107	hsa-miR-3649	12.38	7.89
108	hsa-miR-182-5p	12.37	4.86
109	hsa-miR-744-5p	12.27	1.56
110	hsa-miR-3119	12.27	3.07
111	hsa-miR-27b-5p	12.22	5.95
112	hsa-miR-506-3p	11.42	5.69
113	hsa-miR-3945	11.36	8.09
114	hsa-miR-550a-5p	11.26	2.66
115	hsa-miR-192-5p	11.25	4.57
116	hsa-miR-1185-1-3p	11.14	8.01
117	hsa-miR-6891-5p	11.04	1.54

118	hsa-miR-7848-3p	10.95	3.61
119	hsa-miR-938	10.93	0.11
120	hsa-miR-576-3p	10.71	1.98
121	hsa-miR-6797-5p	10.44	3.00
122	hsa-miR-1277-3p	10.37	6.07
123	hsa-miR-130a-3p	10.33	2.42
124	hsa-miR-4753-5p	9.97	4.81
125	hsa-miR-28-3p	9.95	2.23
126	hsa-miR-3646	9.71	3.27
127	hsa-miR-3614-5p	9.56	2.73
128	hsa-miR-30a-5p	9.31	5.95
129	hsa-miR-6734-3p	9.09	5.50
130	hsa-miR-5694	8.78	5.46
131	hsa-miR-4484	8.77	1.69
132	hsa-miR-4713-3p	8.66	6.60
133	hsa-miR-802	8.28	5.03
134	hsa-miR-4750-5p	8.23	2.07
135	hsa-miR-371b-5p	8.22	2.23
136	hsa-miR-4754	8.14	2.43
137	hsa-miR-6835-3p	8.13	5.24
138	hsa-miR-5089-5p	8.07	0.21
139	hsa-miR-6855-5p	7.86	3.06
140	hsa-miR-1197	7.80	3.04
141	hsa-miR-6132	7.71	3.87
142	hsa-miR-6784-3p	7.54	5.37
143	hsa-miR-30d-5p	7.49	0.78
144	hsa-miR-484	7.42	0.23
145	hsa-miR-372-3p	7.41	2.36
146	hsa-miR-221-5p	7.41	1.06
147	hsa-miR-4788	7.08	3.38
148	hsa-miR-3682-3p	6.89	5.00
149	hsa-miR-2681-3p	6.83	4.01
150	hsa-miR-6722-5p	6.79	2.45
151	hsa-miR-4459	6.76	3.56
152	hsa-miR-181c-3p	6.69	4.07
153	hsa-miR-3943	6.50	2.58
154	hsa-miR-1912	6.42	1.62
155	hsa-miR-1306-3p	6.33	2.22
156	hsa-miR-6772-5p	6.31	4.49
157	hsa-miR-1266-5p	6.20	1.57
158	hsa-miR-6720-5p	6.20	3.37

159	hsa-miR-4746-5p	6.08	3.60
160	hsa-miR-7-2-3p	6.05	1.94
161	hsa-miR-541-3p	5.99	2.25
162	hsa-miR-6798-5p	5.97	1.90
163	hsa-miR-6840-3p	5.79	4.47
164	hsa-miR-4695-3p	5.68	2.31
165	hsa-miR-6743-5p	5.66	1.36
166	hsa-miR-409-5p	5.59	4.33
167	hsa-miR-4700-5p	5.56	2.45
168	hsa-miR-3161	5.53	2.31
169	hsa-miR-5196-3p	5.44	1.36
170	hsa-miR-5010-5p	5.42	3.40
171	hsa-miR-7702	5.39	2.73
172	hsa-miR-3121-3p	5.14	2.93
173	hsa-miR-7977	5.10	1.29
174	hsa-miR-4786-5p	5.08	2.33
175	hsa-miR-25-3p	4.94	2.32
176	hsa-miR-361-5p	4.84	2.21
177	hsa-miR-6854-5p	4.84	2.58
178	hsa-miR-937-3p	4.82	0.74
179	hsa-miR-4516	4.77	1.29
180	hsa-miR-3691-5p	4.76	1.57
181	hsa-miR-30e-5p	4.71	1.90
182	hsa-miR-1238-5p	4.69	1.48
183	hsa-miR-3611	4.60	0.37
184	hsa-miR-381-5p	4.50	2.03
185	hsa-miR-370-3p	4.49	0.46
186	hsa-miR-410-5p	4.49	1.29
187	hsa-miR-128-1-5p	4.37	0.45
188	hsa-miR-8078	4.37	0.73
189	hsa-miR-425-5p	4.28	0.92
190	hsa-miR-6868-5p	4.27	2.18
191	hsa-miR-4461	4.15	2.24
192	hsa-miR-1909-3p	4.11	2.15
193	hsa-miR-640	4.11	1.72
194	hsa-miR-3914	4.10	0.22
195	hsa-miR-3196	4.05	1.49
196	hsa-miR-92a-3p	4.02	2.03
197	hsa-miR-186-5p	3.97	1.14
198	hsa-miR-3690	3.76	0.46
199	hsa-miR-762	3.71	0.80

200	hsa-miR-542-3p	3.68	0.78
201	hsa-miR-3200-5p	3.67	0.31
202	hsa-miR-9-3p	3.63	2.38
203	hsa-miR-922	3.61	0.49
204	hsa-miR-3944-3p	3.50	1.30
205	hsa-miR-6739-5p	3.46	0.40
206	hsa-miR-1249	3.37	0.96
207	hsa-miR-3122	3.34	0.37
208	hsa-miR-181c-5p	3.34	0.54
209	hsa-miR-6086	3.33	0.64
210	hsa-miR-891a-5p	3.29	1.07
211	hsa-miR-6826-5p	3.22	1.74
212	hsa-miR-6079	3.19	1.22
213	hsa-miR-1243	3.14	1.59
214	hsa-miR-7107-5p	3.02	1.05
215	hsa-miR-4798-5p	2.90	0.97
216	hsa-miR-8058	2.85	0.31
217	hsa-miR-761	2.78	1.67
218	hsa-miR-6757-5p	2.77	1.53
219	hsa-miR-6839-5p	2.74	0.59
220	hsa-miR-5197-3p	2.68	0.80
221	hsa-miR-1253	2.68	0.37
222	hsa-miR-4515	2.68	1.11
223	hsa-miR-3147	2.65	1.55
224	hsa-miR-6816-3p	2.57	0.65
225	hsa-miR-130b-5p	2.46	0.28
226	hsa-miR-128-3p	2.44	0.93
227	hsa-miR-1286	2.43	0.89
228	hsa-miR-3181	2.38	1.09
229	hsa-miR-7847-3p	2.38	0.88
230	hsa-miR-1273c	2.38	0.61
231	hsa-miR-92b-3p	2.32	0.61
232	hsa-miR-769-3p	2.29	0.60
233	hsa-miR-6809-5p	2.27	0.48
234	hsa-miR-143-3p	2.24	1.24
235	hsa-miR-5693	2.20	0.54
236	hsa-miR-6124	2.13	0.47
237	hsa-miR-219b-3p	2.12	0.63
238	hsa-miR-26b-5p	2.12	0.29
239	hsa-miR-7161-3p	2.02	0.56
