# Supplementary material for: N6-Adenosine Methylation in MiRNAs
Source: PLoS One. 2015 Feb 27;10(2):e0118438. doi: 10.1371/journal.pone.0118438 (PMC4344304; doi:10.1371/journal.pone.0118438)
Supplement: S4 Table — (PDF) [file pone.0118438.s004.pdf]

Supplementary Table 4.

List of the 46 motifs (3-mers) found to be significantly overrepresented in the immunoprecipitated miRNAs in comparison to the remaining miRNAs, as reported by MoSDi, sorted by score (decreasing).

	**motif**	**score (negative base-10 logarithm of p-value)**	**number of sequences with the motif column**	**p value**
1	RAA	7.31	119	4.90E-08
2	RAM	7.2936	170	5.09E-08
3	RAH	7.158	202	6.95E-08
4	YGA	7.0113	125	9.74E-08
5	RAY	6.9782	186	1.05E-07
6	AAS	6.675	128	2.11E-07
7	TGA	6.3849	110	4.12E-07
8	GAH	6.3417	175	4.55E-07
9	RAW	6.2971	166	5.05E-07
10	HMG	6.2516	223	5.60E-07
11	YRA	6.2508	175	5.61E-07
12	AAG	6.0456	94	9.00E-07
13	DAA	5.9434	134	1.14E-06
14	AAV	5.8723	136	1.34E-06
15	VAA	5.8523	140	1.41E-06
16	GAW	5.7734	137	1.69E-06
17	BGA	5.7571	187	1.75E-06
18	GAY	5.5561	135	2.78E-06
19	GAM	5.5468	139	2.84E-06
20	AHG	5.527	156	2.97E-06
21	AWS	5.4874	186	3.26E-06
22	AMG	5.4152	109	3.84E-06
23	HGA	5.4121	165	3.87E-06
24	SAA	5.276	129	5.30E-06
25	AWG	5.2554	143	5.55E-06
26	AAM	5.2071	96	6.21E-06
27	AAK	5.1878	127	6.49E-06
28	AAB	5.1782	152	6.63E-06
29	ARA	5.1146	131	7.68E-06
30	RAC	5.0676	119	8.56E-06
31	RAR	5.0319	186	9.29E-06
32	AAR	4.9595	112	1.10E-05

33	GAA	4.9479	88	1.13E-05
34	WGA	4.89	155	1.29E-05
35	WRA	4.8774	183	1.33E-05
36	AMK	4.8766	179	1.33E-05
37	TRA	4.8728	132	1.34E-05
38	AWV	4.8715	197	1.34E-05
39	KGA	4.8651	177	1.36E-05
40	MRA	4.8569	159	1.39E-05
41	WAG	4.847	136	1.42E-05
42	HRA	4.7917	200	1.62E-05
43	GAD	4.7842	185	1.64E-05
44	SRA	4.7651	184	1.72E-05
45	WMG	4.755	162	1.76E-05
46	MGA	4.7094	113	1.95E-05
