# Supplementary material for: N6-Adenosine Methylation in MiRNAs
Source: PLoS One. 2015 Feb 27;10(2):e0118438. doi: 10.1371/journal.pone.0118438 (PMC4344304; doi:10.1371/journal.pone.0118438)
Supplement: S5 Table — (PDF) [file pone.0118438.s005.pdf]

**Supplementary Table 5.**

List of the top 50 motifs (4-mers) found to be significantly overrepresented in the immunoprecipitated miRNAs in comparison to the remaining miRNAs, as reported by MoSDi, sorted by score (decreasing).

| | **motif** | **score (negative base-10 logarithm of p-value)** | **number of sequences with the motif column** | **p value** |
|---|---|---|---|---|
| 1 | ADRA | 16.717 | 133 | 1.92E-17 |
| 2 | ANRA | 15.3112 | 139 | 4.88E-16 |
| 3 | ABRA | 14.0125 | 124 | 9.72E-15 |
| 4 | WVAA | 13.9105 | 113 | 1.23E-14 |
| 5 | AKRA | 13.719 | 112 | 1.91E-14 |
| 6 | WRAA | 13.6199 | 94 | 2.40E-14 |
| 7 | AWRA | 13.52 | 83 | 3.02E-14 |
| 8 | ADVA | 13.2212 | 154 | 6.01E-14 |
| 9 | AHRA | 13.0621 | 95 | 8.67E-14 |
| 10 | MBRA | 12.9809 | 166 | 1.04E-13 |
| 11 | AWVA | 12.936 | 104 | 1.16E-13 |
| 12 | MBGA | 12.8053 | 129 | 1.57E-13 |
| 13 | ANVA | 12.4478 | 165 | 3.57E-13 |
| 14 | MNRA | 11.9903 | 184 | 1.02E-12 |
| 15 | HRAA | 11.8907 | 104 | 1.29E-12 |
| 16 | HVAA | 11.8671 | 122 | 1.36E-12 |
| 17 | ADRM | 11.7492 | 174 | 1.78E-12 |
| 18 | WNAA | 11.5685 | 117 | 2.70E-12 |
| 19 | WDAA | 11.4052 | 100 | 3.93E-12 |
| 20 | ANDA | 11.3384 | 154 | 4.59E-12 |
| 21 | ARRA | 11.2592 | 107 | 5.51E-12 |
| 22 | HRAR | 11.1588 | 166 | 6.94E-12 |
| 23 | ABDA | 11.1446 | 141 | 7.17E-12 |
| 24 | AWGA | 11.1345 | 59 | 7.34E-12 |
| 25 | ARAA | 11.1249 | 65 | 7.50E-12 |
| 26 | MDRA | 11.1079 | 173 | 7.80E-12 |
| 27 | AHVA | 11.0628 | 120 | 8.65E-12 |
| 28 | ADDA | 11.0355 | 142 | 9.22E-12 |
| 29 | AWVR | 10.9682 | 150 | 1.08E-11 |
| 30 | ADGA | 10.9342 | 94 | 1.16E-11 |
| 31 | RRAH | 10.8211 | 168 | 1.51E-11 |
| 32 | AHGA | 10.7953 | 62 | 1.60E-11 |

| | | | | |
|---|---|---|---|---|
| 33 | ANGM | 10.7581 | 142 | 1.75E-11 |
| 34 | AAVR | 10.748 | 99 | 1.79E-11 |
| 35 | ABVA | 10.7286 | 149 | 1.87E-11 |
| 36 | ATGA | 10.7159 | 38 | 1.92E-11 |
| 37 | AVAA | 10.705 | 76 | 1.97E-11 |
| 38 | ANGA | 10.6814 | 96 | 2.08E-11 |
| 39 | RVAY | 10.6682 | 181 | 2.15E-11 |
| 40 | ADAA | 10.6132 | 71 | 2.44E-11 |
| 41 | AYRA | 10.5987 | 66 | 2.52E-11 |
| 42 | MNGA | 10.5161 | 151 | 3.05E-11 |
| 43 | WDRA | 10.4974 | 172 | 3.18E-11 |
| 44 | AVRA | 10.4304 | 115 | 3.71E-11 |
| 45 | MYGA | 10.3841 | 86 | 4.13E-11 |
| 46 | ARVA | 10.3684 | 129 | 4.28E-11 |
| 47 | AHGM | 10.3351 | 94 | 4.62E-11 |
| 48 | AYGA | 10.3033 | 42 | 4.97E-11 |
| 49 | MKGA | 10.255 | 117 | 5.56E-11 |
| 50 | ADVM | 10.2517 | 199 | 5.60E-11 |
