# Supplementary material for: N6-Adenosine Methylation in MiRNAs
Source: PLoS One. 2015 Feb 27;10(2):e0118438. doi: 10.1371/journal.pone.0118438 (PMC4344304; doi:10.1371/journal.pone.0118438)
Supplement: S6 Table — (PDF) [file pone.0118438.s006.pdf]

Supplementary Table 6.

List of the top 50 motifs (5-mers) found to be significantly overrepresented in the immunoprecipitated miRNAs in comparison to the remaining miRNAs, as reported by MoSDi, sorted by score (decreasing).

	**motif**	**score (negative base-10 logarithm of p-value)**	**number of sequences with the motif column**	**p value**
1	ADRAB	19.3887	122	4.09E-20
2	ADRAN	16.8124	129	1.54E-17
3	AKRAB	16.8053	99	1.57E-17
4	ANRAB	15.8759	124	1.33E-16
5	NADRA	15.852	127	1.41E-16
6	DADRA	15.7829	104	1.65E-16
7	ADRAK	15.657	95	2.20E-16
8	AHRAD	15.5798	87	2.63E-16
9	ADRAY	15.5017	83	3.15E-16
10	ARRAB	15.1926	101	6.42E-16
11	DAKRA	15.0755	91	8.40E-16
12	AKRAY	15.0183	67	9.59E-16
13	ADVAK	14.8974	117	1.27E-15
14	RWWGV	14.8926	141	1.28E-15
15	DABRA	14.7325	101	1.85E-15
16	RABRA	14.7159	84	1.92E-15
17	AWVAD	14.6676	91	2.15E-15
18	RHRAA	14.6476	72	2.25E-15
19	DANRA	14.6115	111	2.45E-15
20	RANRA	14.6022	93	2.50E-15
21	ANRAD	14.5775	118	2.65E-15
22	ADRAH	14.4975	100	3.18E-15
23	RADRA	14.4649	86	3.43E-15
24	GWDRA	14.4193	101	3.81E-15
25	AWRAK	14.3876	61	4.10E-15
26	AWRAD	14.3796	72	4.17E-15
27	WRAAB	14.3347	86	4.63E-15
28	RWHGV	14.3084	151	4.92E-15
29	AWVAK	14.2797	78	5.25E-15
30	WVAAN	14.2215	111	6.01E-15
31	AHRVR	14.2158	136	6.08E-15
32	RWRAA	14.2157	61	6.09E-15
33	AHRAR	14.1776	70	6.64E-15

34	VWRRA	14.175	144	6.68E-15
35	DADRM	14.1423	146	7.21E-15
36	RAHRR	14.1014	115	7.92E-15
37	ANRAK	14.1004	100	7.94E-15
38	RWVAA	14.0829	72	8.26E-15
39	RHRRA	14.0748	131	8.42E-15
40	ADRMK	14.0539	127	8.83E-15
41	ADGMK	14.0458	96	9.00E-15
42	RAKRA	14.0079	75	9.82E-15
43	ABRAB	13.9731	104	1.06E-14
44	KADRA	13.948	88	1.13E-14
45	AHRRR	13.9477	117	1.13E-14
46	WVAAB	13.9362	100	1.16E-14
47	ABRAN	13.886	121	1.30E-14
48	AKRAN	13.8753	109	1.33E-14
49	VHRAA	13.8705	92	1.35E-14
50	WRRAB	13.8583	147	1.39E-14
