# Supplementary material for: N6-Adenosine Methylation in MiRNAs
Source: PLoS One. 2015 Feb 27;10(2):e0118438. doi: 10.1371/journal.pone.0118438 (PMC4344304; doi:10.1371/journal.pone.0118438)
Supplement: S7 Table — (PDF) [file pone.0118438.s007.pdf]

Supplementary Table 7.

List of the top 50 motifs (3-mers) found to be discriminating between immunoprecipitated miRNAs and remaining miRNAs by Fisher's exact test on the number of sequences with and without the motif in immunoprecipitated and remaining miRNAs.

	motif	Fisher p-value	% of IPed miRNAs with motif	% of remaining miRNAs with motif	% of all miRNAs with motif	ratio (IPed/remaining)	difference (IPed-remaining)
1	AAB	4.71E-06	63.60%	48.00%	49.40%	1.326	15.6
2	AAD	1.42E-05	56.10%	41.40%	42.70%	1.355	14.7
3	AAG	1.11E-05	39.30%	25.60%	26.90%	1.536	13.7
4	AAH	1.65E-05	50.20%	35.80%	37.10%	1.403	14.4
5	AAS	5.53E-06	53.60%	38.10%	39.50%	1.406	15.5
6	AAV	5.34E-06	56.90%	41.50%	42.90%	1.373	15.5
7	ABR	5.68E-07	89.50%	76.10%	77.30%	1.177	13.5
8	AGR	5.13E-07	70.70%	53.90%	55.50%	1.311	16.8
9	AHD	2.11E-06	91.20%	79.10%	80.20%	1.153	12.1
10	AHG	9.16E-06	65.30%	50.30%	51.70%	1.299	15
11	AHK	1.56E-05	85.80%	73.40%	74.60%	1.169	12.4
12	AKV	1.50E-05	92.10%	81.60%	82.60%	1.128	10.4
13	AMB	8.49E-06	83.30%	70.10%	71.30%	1.188	13.2
14	AMD	1.46E-06	82.40%	67.70%	69.10%	1.217	14.7
15	AMK	1.39E-06	74.90%	59.20%	60.60%	1.266	15.7
16	ARA	3.10E-06	54.80%	39.00%	40.50%	1.406	15.8
17	ARD	1.57E-05	87.90%	76.00%	77.10%	1.156	11.9
18	ARR	2.31E-07	81.60%	65.70%	67.20%	1.242	15.9
19	ASD	5.55E-08	95.00%	82.60%	83.80%	1.15	12.4
20	ASK	4.19E-06	87.00%	74.20%	75.40%	1.173	12.8
21	ASR	6.44E-07	81.60%	66.20%	67.70%	1.232	15.4
22	AVD	1.21E-06	96.20%	86.20%	87.20%	1.116	10
23	AVG	7.43E-06	70.70%	55.80%	57.20%	1.267	14.9
24	AVK	1.07E-07	93.70%	81.00%	82.20%	1.158	12.8
25	AVR	2.22E-06	87.90%	74.70%	76.00%	1.176	13.1
26	AWB	1.05E-05	83.70%	70.70%	71.90%	1.183	13
27	AWG	1.61E-05	59.80%	45.00%	46.40%	1.33	14.8
28	AWS	3.47E-06	77.80%	63.00%	64.30%	1.236	14.9
29	AWV	2.71E-07	82.40%	66.80%	68.30%	1.234	15.6
30	AYD	1.55E-05	83.30%	70.40%	71.60%	1.183	12.9
31	BBT	4.36E-07	83.70%	93.80%	92.80%	0.892	-10.1
32	BGA	1.06E-09	78.20%	58.60%	60.40%	1.335	19.7

33	BGR	5.12E-07	91.20%	78.40%	79.60%	1.164	12.8
34	BKT	2.74E-06	65.30%	79.10%	77.80%	0.825	-13.8
35	BKY	3.89E-07	90.80%	97.80%	97.10%	0.929	-7
36	BRA	1.14E-08	90.00%	74.50%	76.00%	1.207	15.4
37	BTY	6.64E-06	62.30%	76.30%	75.00%	0.818	-13.9
38	DAA	5.46E-07	56.10%	39.10%	40.70%	1.433	16.9
39	DAH	7.71E-09	90.00%	74.30%	75.80%	1.211	15.6
40	DAM	1.30E-06	77.80%	62.30%	63.80%	1.248	15.5
41	DAW	1.98E-07	76.60%	59.70%	61.30%	1.283	16.9
42	DAY	3.93E-08	84.10%	67.60%	69.10%	1.245	16.5
43	DGA	5.98E-09	82.80%	65.00%	66.70%	1.274	17.8
44	DRA	1.39E-07	88.70%	74.30%	75.60%	1.194	14.4
45	DSA	3.35E-07	97.10%	87.00%	87.90%	1.116	10.1
46	GAA	1.32E-06	36.80%	22.20%	23.60%	1.656	14.6
47	GAB	4.88E-08	76.20%	58.30%	60.00%	1.306	17.9
48	GAD	4.02E-09	77.40%	58.30%	60.10%	1.327	19.1
49	GAH	1.51E-10	73.20%	51.90%	53.90%	1.411	21.3
50	GAK	3.37E-06	64.40%	48.60%	50.10%	1.327	15.9
