# Supplementary material for: N6-Adenosine Methylation in MiRNAs
Source: PLoS One. 2015 Feb 27;10(2):e0118438. doi: 10.1371/journal.pone.0118438 (PMC4344304; doi:10.1371/journal.pone.0118438)
Supplement: S8 Table — (PDF) [file pone.0118438.s008.pdf]

Supplementary Table 8.

List of the top 50 motifs (4-mers) found to be discriminating between immunoprecipitated miRNAs and remaining miRNAs by Fisher's exact test on the number of sequences with and without the motif in immunoprecipitated and remaining miRNAs.

	motif	Fisher p-value	% of IPed miRNAs with motif	% of remaining miRNAs with motif	% of all miRNAs with motif	ratio (IPed/remaining)	difference (IPed-remaining)
1	AABR	2.60E-07	41.80%	25.70%	27.20%	1.629	16.2
2	AAGV	6.92E-07	32.20%	18.00%	19.30%	1.789	14.2
3	AAND	9.30E-07	57.30%	40.70%	42.30%	1.408	16.6
4	AANR	1.14E-06	47.70%	31.70%	33.20%	1.505	16
5	AARR	1.05E-06	35.10%	20.70%	22.00%	1.699	14.5
6	AASR	6.22E-07	33.10%	18.70%	20.00%	1.772	14.4
7	AASV	8.11E-07	42.70%	27.00%	28.50%	1.581	15.7
8	AAVR	3.69E-07	41.40%	25.50%	27.00%	1.623	15.9
9	ABAA	1.54E-07	26.40%	12.90%	14.20%	2.042	13.4
10	ABAH	8.79E-07	50.60%	34.30%	35.80%	1.477	16.3
11	ABAW	2.24E-07	41.80%	25.50%	27.00%	1.642	16.4
12	ABDA	5.43E-12	59.00%	35.70%	37.90%	1.652	23.3
13	ABDM	8.22E-08	77.80%	60.70%	62.30%	1.283	17.2
14	ABGA	1.47E-09	34.30%	17.00%	18.60%	2.017	17.3
15	ABGM	1.01E-08	49.40%	30.50%	32.30%	1.617	18.8
16	ABNA	1.65E-10	67.80%	46.20%	48.20%	1.469	21.6
17	ABNW	4.41E-08	89.50%	74.70%	76.00%	1.199	14.9
18	ABRA	1.28E-14	51.90%	26.90%	29.20%	1.929	25
19	ABRH	8.98E-09	79.50%	61.20%	62.90%	1.298	18.3
20	ABRM	4.04E-11	68.60%	46.30%	48.40%	1.482	22.3
21	ABRN	9.59E-07	88.30%	74.80%	76.00%	1.181	13.5
22	ABRW	6.37E-08	66.50%	48.20%	49.90%	1.381	18.3
23	ABSA	1.07E-06	47.30%	31.30%	32.80%	1.51	16
24	ABVA	7.75E-12	62.30%	39.20%	41.40%	1.59	23.1
25	ABVM	9.51E-08	80.80%	64.10%	65.70%	1.259	16.6
26	ABVW	2.68E-08	82.80%	65.80%	67.40%	1.259	17
27	ADAA	6.96E-09	29.70%	14.20%	15.70%	2.085	15.5
28	ADAH	1.97E-07	51.90%	34.50%	36.10%	1.504	17.4
29	ADAM	1.35E-07	41.80%	25.20%	26.80%	1.659	16.6
30	ADAR	2.40E-07	44.80%	28.20%	29.70%	1.588	16.6
31	ADAV	9.14E-07	54.00%	37.40%	38.90%	1.443	16.6
32	ADAW	2.03E-07	41.40%	25.10%	26.70%	1.648	16.3

33	ADDA	4.00E-12	59.40%	36.10%	38.30%	1.646	23.3
34	ADDM	1.66E-07	77.80%	61.10%	62.60%	1.275	16.8
35	ADGA	2.35E-10	39.30%	20.30%	22.10%	1.938	19
36	ADGM	7.47E-10	56.90%	36.10%	38.10%	1.575	20.8
37	ADNA	2.60E-10	66.90%	45.60%	47.60%	1.47	21.4
38	ADRA	3.17E-15	55.60%	29.60%	32.10%	1.879	26
39	ADRM	1.58E-11	72.80%	50.30%	52.40%	1.447	22.5
40	ADRR	5.76E-08	70.70%	52.50%	54.20%	1.346	18.2
41	ADSA	2.02E-09	52.70%	32.70%	34.60%	1.611	20
42	ADSM	3.11E-08	73.60%	55.30%	57.00%	1.331	18.3
43	ADVA	1.10E-12	64.40%	40.20%	42.50%	1.603	24.2
44	ADVM	1.55E-09	83.30%	64.60%	66.40%	1.288	18.6
45	ADVR	4.39E-07	78.20%	62.10%	63.60%	1.26	16.2
46	ADVW	1.76E-07	81.20%	64.90%	66.50%	1.25	16.2
47	ADWA	2.44E-07	38.10%	22.30%	23.80%	1.706	15.8
48	AGAA	5.19E-07	17.60%	7.10%	8.10%	2.482	10.5
49	AGAH	4.17E-08	34.70%	18.80%	20.30%	1.849	15.9
50	AGAM	2.32E-07	26.80%	13.40%	14.60%	2.001	13.4
