# Supplementary material for: N6-Adenosine Methylation in MiRNAs
Source: PLoS One. 2015 Feb 27;10(2):e0118438. doi: 10.1371/journal.pone.0118438 (PMC4344304; doi:10.1371/journal.pone.0118438)
Supplement: S9 Table — (PDF) [file pone.0118438.s009.pdf]

Supplementary Table 9.

List of the top 50 motifs found to be discriminating between immunoprecipitated miRNAs and remaining miRNAs by Fisher's exact test on the number of sequences with and without the motif in immunoprecipitated and remaining miRNAs.

	motif	Fisher p-value	% of IPed miRNAs with motif	% of remaining miRNAs with motif	% of all miRNAs with motif	ratio (IPed/remaining)	difference (IPed-remaining)
1	AABGA	6.15E-08	11.70%	3.20%	4.00%	3.667	8.5
2	AABRA	4.35E-08	16.70%	5.90%	6.90%	2.85	10.9
3	AABRD	2.04E-08	35.60%	19.20%	20.70%	1.855	16.4
4	AABRN	4.82E-08	41.80%	24.70%	26.30%	1.691	17.1
5	AABRR	5.70E-09	27.60%	12.50%	13.90%	2.205	15.1
6	AABRV	2.20E-08	34.70%	18.50%	20.00%	1.875	16.2
7	AADGA	4.52E-08	14.60%	4.70%	5.70%	3.083	9.9
8	AADGM	4.18E-08	21.80%	9.20%	10.40%	2.366	12.6
9	AADGV	1.19E-08	28.00%	13.20%	14.60%	2.129	14.9
10	AADRA	6.21E-08	18.80%	7.40%	8.50%	2.55	11.4
11	AAKRR	5.70E-08	23.40%	10.40%	11.60%	2.252	13
12	AANGA	9.28E-09	15.50%	4.90%	5.90%	3.173	10.6
13	AANGM	3.03E-09	23.80%	9.70%	11.00%	2.455	14.1
14	AANGR	3.10E-08	23.00%	9.90%	11.20%	2.317	13.1
15	AANGV	1.55E-09	30.10%	14.00%	15.50%	2.153	16.1
16	AANRA	2.43E-08	20.50%	8.10%	9.20%	2.539	12.4
17	AANRD	7.86E-08	41.80%	24.90%	26.50%	1.679	16.9
18	AANRR	2.48E-08	33.10%	17.20%	18.70%	1.919	15.8
19	AANRV	6.67E-08	40.20%	23.40%	25.00%	1.716	16.8
20	AARGM	4.88E-08	18.80%	7.30%	8.40%	2.58	11.5
21	AARGV	6.80E-09	23.40%	9.80%	11.00%	2.401	13.7
22	AARVA	6.86E-08	20.90%	8.70%	9.90%	2.399	12.2
23	AASRR	2.60E-08	22.20%	9.30%	10.50%	2.378	12.8
24	AASRV	3.81E-08	28.50%	13.80%	15.20%	2.059	14.6
25	AASVD	6.56E-08	36.40%	20.40%	21.90%	1.786	16
26	AAVGM	3.58E-09	20.90%	7.90%	9.10%	2.662	13.1
27	AAVGV	1.88E-09	25.50%	10.70%	12.10%	2.384	14.8
28	AAVRD	8.54E-08	36.00%	20.10%	21.60%	1.792	15.9
29	ABDAK	1.32E-09	38.50%	20.40%	22.10%	1.889	18.1
30	ABDAN	4.25E-12	57.70%	34.50%	36.70%	1.672	23.2
31	ABDAS	8.10E-08	38.10%	21.80%	23.30%	1.746	16.3
32	ABDAY	4.67E-08	31.80%	16.60%	18.00%	1.918	15.2

33	ABDMK	1.44E-09	55.20%	34.90%	36.80%	1.581	20.3
34	ABDMN	6.77E-08	76.60%	59.10%	60.70%	1.296	17.5
35	ABDRR	1.86E-08	65.70%	46.50%	48.30%	1.413	19.2
36	ABGAB	1.64E-10	29.30%	12.70%	14.20%	2.315	16.6
37	ABGAD	2.65E-08	28.00%	13.40%	14.80%	2.088	14.6
38	ABGAH	9.89E-11	25.90%	10.20%	11.70%	2.546	15.8
39	ABGAK	2.53E-08	23.00%	9.80%	11.00%	2.348	13.2
40	ABGAN	2.40E-10	33.90%	16.10%	17.80%	2.099	17.7
41	ABGAV	6.04E-08	27.20%	13.10%	14.40%	2.072	14.1
42	ABGAW	4.37E-08	18.80%	7.30%	8.30%	2.596	11.6
43	ABGAY	7.57E-10	19.20%	6.50%	7.70%	2.952	12.7
44	ABGMB	1.89E-10	41.00%	21.50%	23.40%	1.903	19.5
45	ABGMD	1.95E-08	41.00%	23.60%	25.20%	1.736	17.4
46	ABGMH	4.14E-08	38.90%	22.20%	23.80%	1.75	16.7
47	ABGMK	2.18E-09	32.20%	15.50%	17.10%	2.073	16.7
48	ABGMN	1.05E-08	47.70%	29.10%	30.80%	1.639	18.6
49	ABGMY	1.90E-08	29.30%	14.20%	15.70%	2.056	15
50	ABKMK	4.58E-08	43.10%	25.80%	27.40%	1.672	17.3
